# Enzyme-activatable kidney-targeted dendrimer-drug conjugate for efficient childhood nephrotic syndrome therapy

**DOI:** 10.7150/thno.101606

**Published:** 2024-10-21

**Authors:** Danfei Chen, Junjun Xu, Sha Lv, Xiaoqin Jin, Yuyan Chen, Haifang Cai, Qili Wang, Xiaobo Xuan, Guowei Wang, Weidong Fei, Jian Chen

**Affiliations:** 1 Department of Pediatrics, The First Affiliated Hospital of Zhejiang Chinese Medical University (Zhejiang Provincial Hospital of Chinese Medicine), Zhejiang Chinese Medical University, Hangzhou 310006, China.; 2 Department of Pharmacy, The Second Affiliated Hospital of Zhejiang University School of Medicine, Zhejiang University, Hangzhou 310009, China.; 3 Academy of Chinese Medical Sciences, Zhejiang Chinese Medical University, Hangzhou 310053, China.; 4 Research Center of Ultrasound in Medicine and Biomedical Engineering, The Second Affiliated Hospital of Zhejiang University School of Medicine, Zhejiang University, Hangzhou 310009, China.; 5 ZJU-Hangzhou Global Scientific and Technological Innovation Center, Zhejiang University, Hangzhou 311215, China.; 6 Women's Hospital, Zhejiang University School of Medicine, Zhejiang University, Hangzhou 310006, China.

**Keywords:** kidney-targeted drug delivery, childhood nephrotic syndrome, dendrimer-drug conjugate, enzyme-activation, triptolide, *γ*-glutamyl transpeptidase

## Abstract

**Rationale:** Childhood nephrotic syndrome (NS) is a serious disease affecting the health and quality of life of children, which is characterized by a series of pathophysiological changes due to the increased permeability of the glomerular membrane to plasma proteins. Low renal drug distribution and inefficient cellular uptake, resulting from cellular dysfunctions of filtration and internalization, are the main barriers to drug treatment in childhood NS, leading to deterioration in nephropathy. However, efficient therapeutic methods against childhood NS are still lacking in clinic.

**Methods:** This study found that γ-glutamyltransferase (GGT) was highly expressed in the glomeruli of childhood NS in juvenile rats. We proposed GGT as the receptor target of the kidney-targeted drug delivery system, and then designed a GGT enzyme-responsive dendrimer-drug conjugate (GSHPD) as a kidney-targeted drug delivery platform for treating childhood NS. This platform could overcome the physiological and cellular uptake barriers of the kidney through receptor-mediated transcytosis.

**Results:** GSHPD was composed of glutathione-modified polyamidoamine dendrimers and conjugated with triptolide (TP). Once GSHPD was delivered to the glomerulus in nephropathy, the overexpressed GGT in the endothelial cells of the glomerular capillaries activated the γ-glutamyl transfer reactions of glutathione to generate positively charged primary amines. The resulting cationic conjugate rapidly underwent caveola-mediated endocytosis and exocytosis, augmenting its renal accumulation and cellular internalization. Active TP was gradually released by intracellular enzyme hydrolysis, enabling sustained therapeutic effects and resulting in significant recovery of renal physiological function (e.g., lowering the levels of urea nitrogen and serum creatinine, improving the levels of urinary creatinine and creatinine clearance rate, and inhibiting podocyte injury).

**Conclusion:** The conjugate exhibited an excellent kidney-targeted distribution and a potent recovery of renal physiological function in NS of juvenile rats. This study presented a promising and active kidney-targeted drug delivery platform for efficient childhood nephropathy therapy.

## Introduction

Nephrotic syndrome (NS) in children is the second largest renal disease in pediatric clinic. It increases the permeability of the glomerular basement membrane, leading to the loss of a large amount of plasma proteins through urine. The incidence of childhood NS is as high as 4.71/100000, accounting for more than 30% of the total number of hospitalized children with an increasing trend annually 1-4. Among these, refractory NS is considered the most serious and common type of childhood NS. It is characterized by hormone dependence, resistance, and frequent recurrence, which seriously affects the growth of children, eventually developing into chronic renal failure or even death in some cases 1, 4. Unfortunately, no effective radical treatment exists for childhood NS until now.

The etiology and pathogenesis of childhood NS still remain unclear to this day. The clinical manifestations of childhood NS mainly include massive proteinuria, edema, hyperlipidemia, hypoproteinemia, and so forth 1, 5. Currently, immunosuppressants based on hormone therapy, commonly used hormonal drugs (glucocorticoids, e.g., prednisone) and immunosuppressive drugs (alkylating agents, e.g., cyclophosphamide), are recommended for the clinical treatment of childhood NS 1, 2, 4, 6. However, the long-term use of glucocorticoids can result in Cushing's syndrome, metabolic disorders, peptic ulcer, osteoporosis, growth inhibition, and diabetes mellitus. Meanwhile, the use of immunosuppressants may also cause serious systemic adverse reactions, such as allergies, susceptibility to infection, and malignant tumors, which have a great impact on the growth of children, and even was intolerable in some patients, resulting in poor clinical efficacy 7-9.

Herbal preparations or extracts have progressed considerably in treating kidney diseases. Clinically, *Tripterygium* glycoside tablets have been widely used for treating purpura nephritis, NS, diabetic nephropathy, lupus nephritis, and other kidney diseases in Asian countries 10-14. Triptolide (TP), the active ingredient of *Tripterygium* glycosides, had been intensively studied for treating kidney diseases. Pharmacological studies have shown that TP could repair podocytes damage by upregulating the expression of zonula occludens-1 (ZO1), decrease podocyte permeability through Tet methylcytosine dioxygenase 2 (Tet2)-mediated demethylations and regulating expression of miRNA-344b-3p, and inhibit the fibronectin expression, glomerular mesangial cells' hypertrophy and the production of extracellular matrix 13, 15-17. TP could also inhibit the expression of thrombospondin-1, suppress of TGF-β1-Smad2 and p53 pathways, protect the function of intrinsic renal cells, and play a multi-target and multi-level therapeutic role in treating renal diseases 12, 13. However, these active ingredients have poor solubility, short half-life, low renal enrichment rate, poor bioavailability, and strong hepatotoxicity and reproductive toxicity, seriously limiting their clinical application in pediatric patients 18-20. Therefore, a kidney-targeted drug delivery system carrying these effective active ingredients to accurately treat childhood NS by enhancing efficacy and reducing toxicity needs to be urgently developed.

Glomerular capillary endothelial cells (EC), mesangial cells (MC), proximal tubular cells (PTC), podocytes, and myofibroblasts are the main focus of the kidney-targeted drug delivery system 21-24. The clinicopathological characteristics of childhood NS are mainly podocyte actin cytoskeleton rearrangement, EC injury, and MC lesions 25, 26. In addition, PTC lesions, and oversecretion of extracellular matrix in myofibroblasts might also be closely related to the deterioration of renal function in childhood NS 27, 28. The simultaneously targeted therapy of multiple types of renal cells can effectively reverse the occurrence and development of NS in children. The kidney-targeted drug delivery system mainly includes prodrugs, drug-carrier conjugates, drug-loaded nanoparticles, and enzyme-mimicking nanozymes, which can greatly enhance the renal accumulation of drugs and achieve the goal of reducing toxicity 22, 23, 29-31. However, these nanocarriers are not effectively internalized by renal cells due to the damage to renal physiological function and the reduction of cell uptake. Meanwhile, the increase in the degree of fibrosis and the accumulation of extracellular matrix components also form a strong tissue barrier to the penetration of drugs, making it difficult for drugs to spread to all types of pathological cells in kidney tissues. A large number of drugs are easily excreted through the urine without being used. Therefore, the kidney-targeted drug delivery system must overcome the renal physiological and cellular uptake barriers to achieve enhancing efficacy in treating childhood NS.

In multicellular eukaryotic organisms, biomacromolecules are transported across cells (from one side of the cell to the other side of the cell) through transcytosis, completing the in-depth transport of substances 32-34. Vesicles are the main form of transcytosis and have different forms while transporting macromolecular substances. The vesicles initially form caveolae on one side of the cell and then form vesico-vacuolar organelles, passing through the cell. After being transported to the other side, the vesicles fuse with the cell membrane to form a depression again, triggering the exocytosis of macromolecular substances, to achieve transcytosis 34. Cationization can effectively promote the transcytosis of macromolecules and the distribution and penetration of the drug-carrier conjugates deep into the whole tissue. Meanwhile, cations can interact with membrane protein on the cell surface to initiate adhesive binding and promote effective cell uptake 35-37. However, cations are unstable in the blood circulation and easy to agglomerate. Also, they are cleared by immune recognition and the reticuloendothelial system, making kidney accumulation cannot be achieved. Responsive charge reversal is a strategy to solve the charge stability of nanocarriers during drug delivery. The key point of this strategy is to make the carriers negatively charged in the blood circulation. After entering the lesion tissue, it promotes the charge reversal of the nanocarriers under endogenous stimulations (pH, enzyme, reactive oxygen species, and so forth) or exogenous stimulations (magnetic, thermal, optical, acoustic, and so forth) and converts the negative charge into a positive charge 38-41. γ-Glutamyltransferase (GGT) is a cell membrane protease with high content in kidney tissues 42-45. Its role is to transfer glutathione (GSH) and glutamyl moiety on its residues to another peptide or amino acid. In the enzymatic reaction catalyzed by GGT, the glutamyl transfer can promote the change of polypeptide residues from negative to positive, leading to charge reversal. Our study showed that GGT was highly expressed in the renal tissues of juvenile rats with NS and mainly distributed in pathological cells, such as renal capillary EC and podocytes, providing a potential receptor target for constructing the kidney-targeted drug delivery system in childhood NS. Therefore, we proposed that a kidney-targeted drug delivery system mediated by GGT-responsive receptor recognition and transcytosis can overcome the renal physiological and cellular uptake barriers.

**Figure 1:**
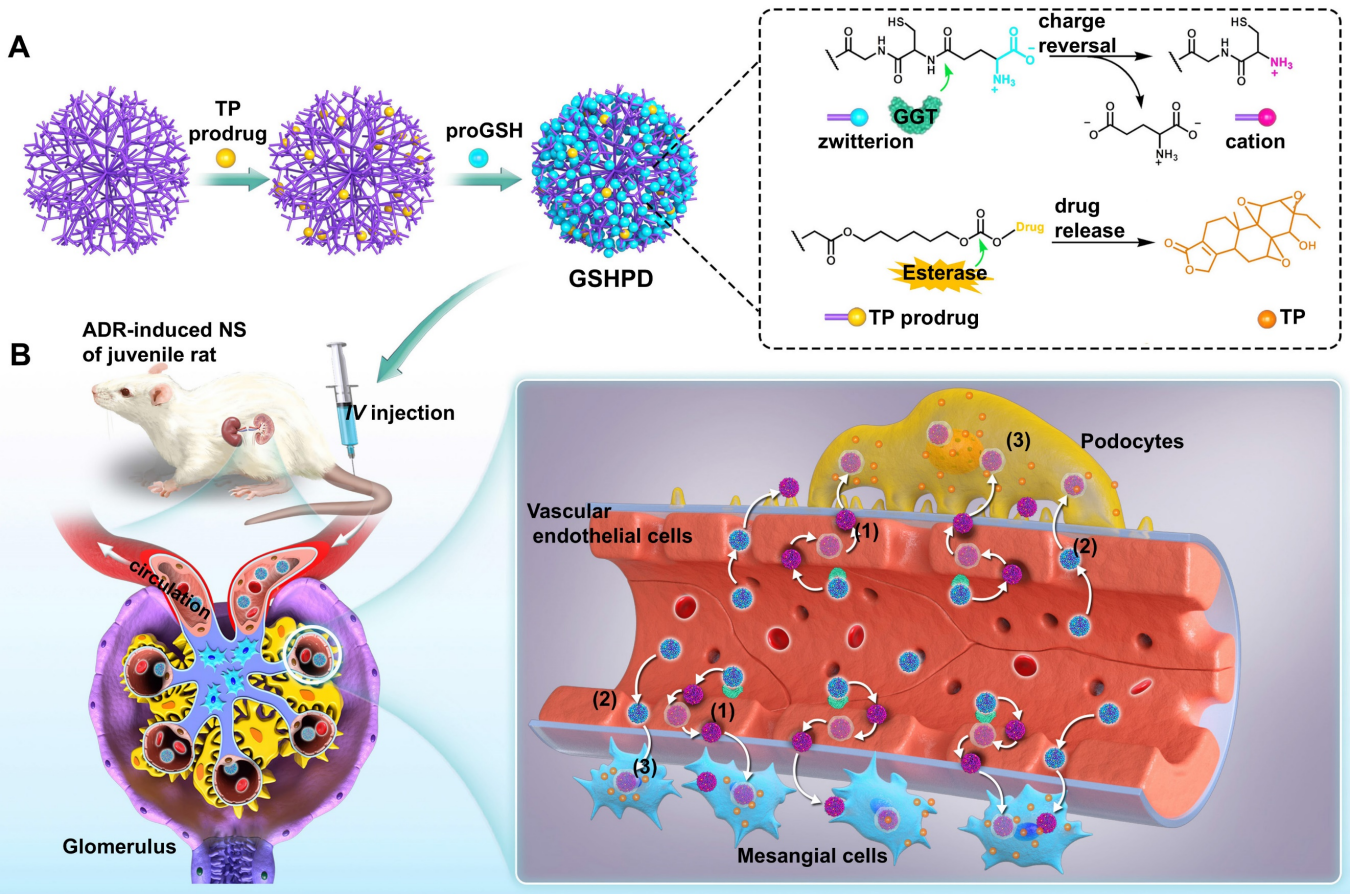
** Schematic representation of *γ*-glutamyl transpeptidase (GGT)-triggered transcytosis of the GSH-modified dendrime-TP conjugate (GSHPD) for hydroxydaunorubicin (ADR)-induced NS therapy in juvenile rats.** (A) Synthesis of GSHPD by conjugating TP to PAMAM dendrimer *via* esterase-cleavable carbonic ester linker and surface modification with GGT-responsive GSH moieties. The proposed reaction mechanism of the charge-switching process by GGT-catalyzed *γ*-glutamyl transfer and TP release by esterase-cleavable linker cleavage. (B) Illustration of the cationization-initiated transcytosis of GSHPD bypassing endothelial cells. After intravenous injection into the ADR-induced juvenile rats, the zwitterionic GSHPD had a prolonged circulation time by inhibiting the nonspecific interactions with blood components. When GSHPD comes in contact with vascular EC in the kidney: *(1)* The anionic GSHPD is converted into a cationic conjugate by the GGT on the membrane of the EC. The cationic charge activates the caveolae-mediated transcytosis of conjugate and facilitates its transport across the endothelium. *(2)* A few of the conjugates can also diffuse into the kidney through extravasation of the fenestrated capillary. *(3)* The conjugate then undergoes fast internalization into kidney cells, where the intracellular esterase triggers the release of TP, improving therapeutic efficacy.

Herein, we developed a GGT-responsive kidney-targeted drug delivery system loaded with TP in the hope of treating childhood NS by enhancing efficiency and reducing toxicity. The G5-generation polyamideamine (PAMAM) dendrimer with precisely controllable particle size was selected as the carrier and GSH (the natural substrate of GGT) as the ligand to construct a GSH-modified TP-conjugated polyamide amine dendrimer (GSHPD). GSHPD is composed of PAMAM chemically coupled with TP prodrug and GSH (**Figure 1A**). GSH can be catalyzed by GGT on the surface of renal cells, such as renal vascular EC, to achieve the cationized-GSHPD. The TP prodrug can release the active TP under the catalysis of renal cell lactonase. After intravenous injection, GSHPD is circulated in the blood to the renal capillary network, and then cationization is achieved by GGT catalytic charge reversal on the surface of vascular EC. The cation induces the transcytosis of GSHPD and enters the renal tissues. Meanwhile, a small amount of GSHPD enters the renal tissues through the vascular openings and vascular windows. Cationization improves GSHPD uptake by renal cells, and GSHPD releases TP *via* the catalysis of intracellular lactonase. The cationic GSHPD diffuses and infiltrates into a variety of cells in renal tissues through continuous transcytosis, improving the therapeutic efficacy of TP (**Figure 1B**).

## Results and Discussion

### Expression and distribution of GGT in juvenile rat NS model

**Figure 2:**
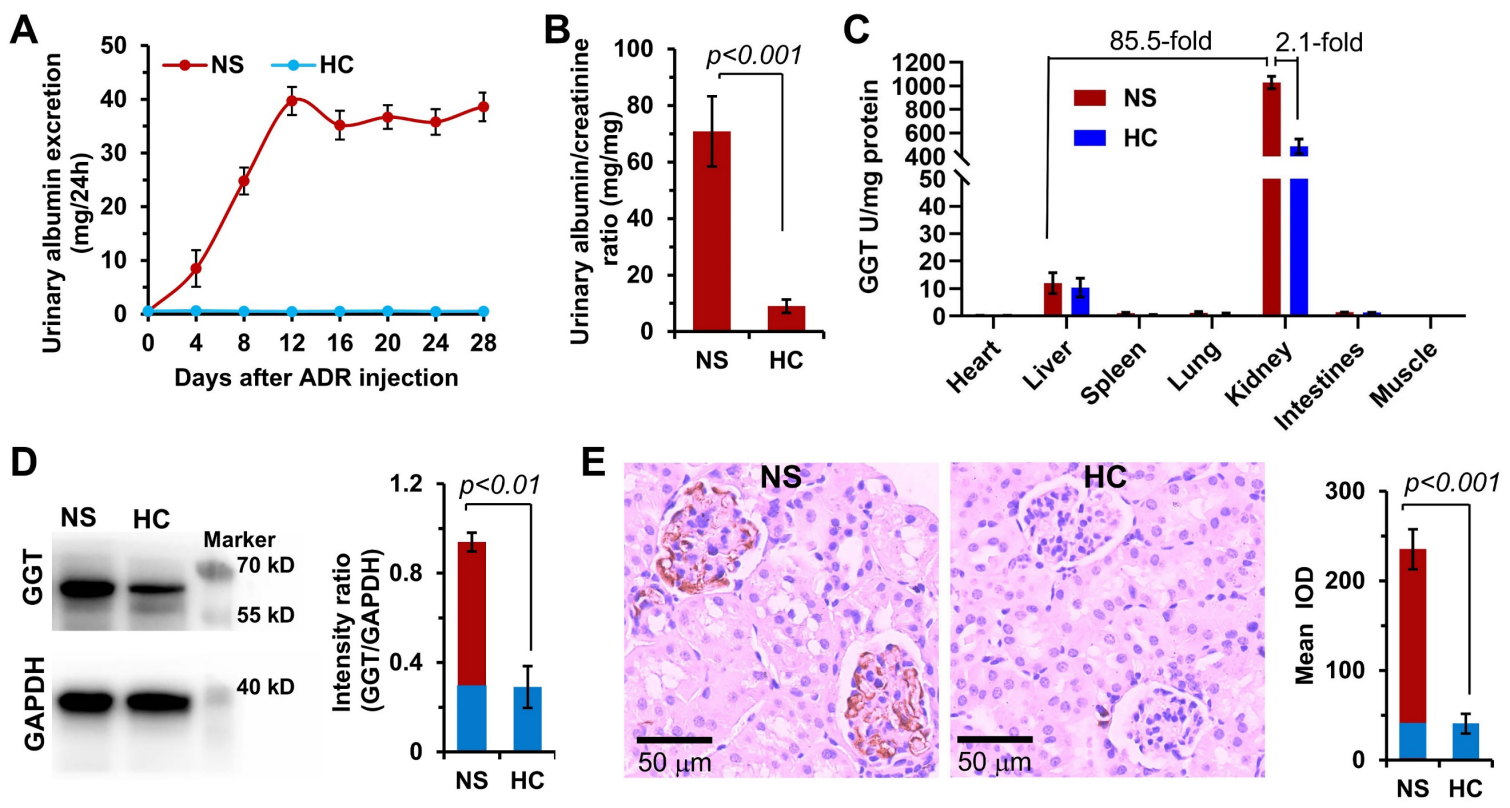
**GGT expression in the ADR-induced NS model of juvenile rats.** (A) Changes in urinary albumin levels in the NS model within 4 weeks after a single high-dose injection of hydroxydaunorubicin (ADR, 10 mg/kg). Urine was collected and measured in a metabolic cage every 4 days. (B) On day 28, the proteinuria index was evaluated (index = proteinuria concentration/creatinine concentration × 100%). (C) GGT level in the major organ tissues of NS rat model. (D) Expression and quantitative analysis of GGT in kidney tissues were determined using Western blotting assay. (E) Expression distribution and quantitative analysis of GGT in kidney tissues were investigated using immunohistochemical assay.

Although high expression of GGT has been reported in liver and kidney tissues of healthy body, the expression of GGT in specific types of diseased kidneys and the distribution characteristics in the kidney are not yet clear. No study has reported on the GGT expression in the childhood NS. Therefore, this study was novel in detecting the expression of GGT in organs and tissues of the ADR-induced NS model of juvenile rat. In this study, 4-week-old male SD rats were intravenously injected with ADR (10 mg/kg) in a single dose. Urine was collected from the rats in metabolic cages every 4 days to determine the urinary protein (UP) and urinary creatinine (UCr) levels. The success of the modeling was determined based on the UP level of >10 mg/24 h and the proteinuria index of >20 46, 47. As shown in **Figure 2A**, the UP level gradually increased after injecting ADR into juvenile rats, reached the highest value on day 12, and then remained basically stable. On day 28, the proteinuria index was as high as 70 in the NS group compared with the healthy control (HC) group, indicating the successful establishment of the ADR-induced NS model (**Figure 2B**). The GGT activity detection kit was used to determine the relative GGT level in various organs and tissues of juvenile rats in the HC and NS groups (**Figure 2C**). The results revealed an extremely high GGT level in the kidneys of NS rats, 85.5 times that in the liver, and 2.1 times that in the HC rats. Subsequently, the expression and distribution of GGT in renal tissues were determined with Western blotting and immunohistochemical analysis (**Figure 2D**). The Western blotting results showed that the expression of GGT was significantly higher in the kidneys of NS rats compared with HC rats. The immunohistochemical analysis indicated that GGT was mainly distributed in glomerular capillary EC and podocytes (**Figure 2E**), which was confirmed using the immunofluorescence co-localization analysis (**Figure S1**). The aforementioned experimental data showed that GGT was highly expressed in glomerular capillary EC and podocytes of juvenile rats with NS. These findings provided a potential therapeutic target for the subsequent design of a kidney-targeted drug delivery system for treating childhood NS.

### Preparation and characterization of conjugates

**Figure 3:**
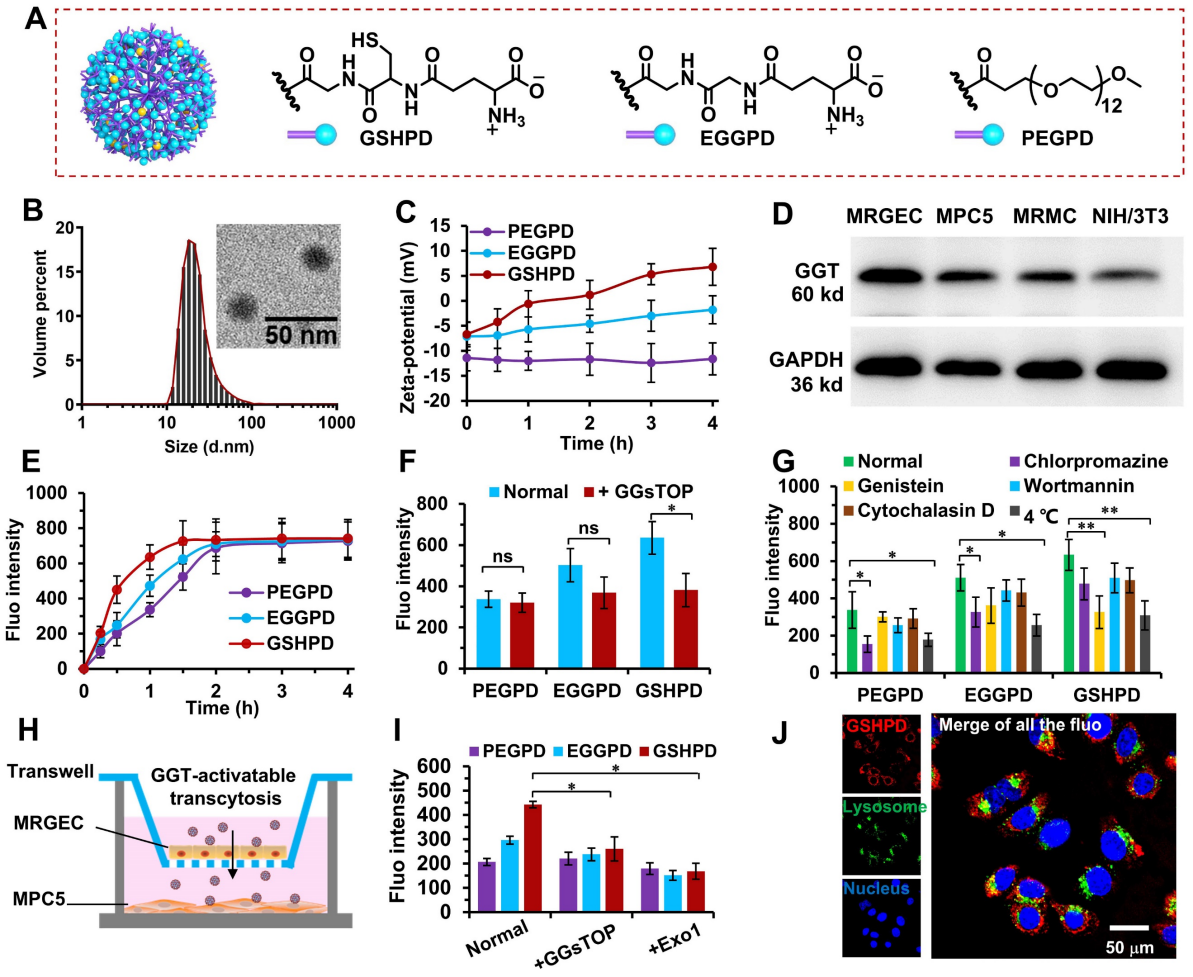
** Chemical structure, characterization, GGT-responsive charge reversal, *in vitro* cellular uptake, endocytosis pathway, and subcellular distribution of the conjugates.** (A) Chemical structure of the conjugates: GSHPD, EGGPD, and PEGPD. (B) Size distribution and TEM image of GSHPD. (C) Changes in the zeta potential of conjugates after incubation in 10 U/mL of GGT enzyme solution within 4 h. (D) GGT expression in the cells of mouse podocyte clone-5 (MPC5), mouse glomerulus endothelial cells (MRGECs), mouse renal mesangial cells (MRMCs), and control cells of mouse embryonic fibroblasts (NIH/3T3). (E) Cellular uptake in the time-dependent manner of the conjugates within 4 h. (F) Inhibitory effects of the GGT inhibitor GGsTOP on cellular uptake after 1 h of incubation. (G) Inhibitory effects of endocytic inhibitors and low temperature (4 ºC) on cellular uptake after 1 h of incubation. (H) Transwell kit designed to investigate the renal vascular permeability using multicellular co-incubation of MRGECs and MPC5 cells. (I) Evaluation of conjugates crossing the renal blood vessels and internalized into MPC5. The mean fluorescence (fluo) intensity of the basolateral medium and MPC5 was measured using a microplate reader. An exocytic pathway inhibitor of EXO1 was also used to test transcytosis properties. (J) Subcellular distribution of GSHPD^Cy5^ (red) after 2 h visualized using CLSM, as simultaneously stained nuclei (blue) and lysosomes (green). Scale bar = 50 μm. Comparison between the two indexed groups: ^*^P < 0.05, ^**^P < 0.01.

GSH, as a natural substrate of GGT, was selected as a ligand-modified drug delivery carrier. Compared with other substrates, GSH had stronger activity and specificity in the process of receptor-ligand interaction 48-50. We coupled TP in prodrug form to the G5-generation PAMAM dendrimer to achieve intracellular drug release and avoid the toxic and side effects caused by early drug release in the blood circulation. This coupling was connected by the esterase-responsive carbonate linker to ensure that the drug delivery system released the drug only after entering the target tissue cells. TP was coupled to PAMAM through the 6-hydroxyhexyl acrylate carbonate and the Michael addition reaction (**Figure S2~S4**). Each PAMAM was coupled with about 12 TP molecules to obtain the PAMAM-TP conjugate. Then, PAMAM-TP respectively reacted with Boc-γ-glu(otbu)-Cys(Trt)-Gly (pro-GSH), Boc-γ-glu(otbu)-Gly-Gly (pro-EGG), and PEG-NHS to obtain the GSH-modified PAMAM-TP-conjugated polyamide amine dendrimer (GSHPD), EGG-modified PAMAM-TP-conjugated polyamide amine dendrimer (EGGPD), PEG-modified PAMAM-TP-conjugated polyamide amine dendrimer (PEGPD) (structure shown in **Figure 3A, and Figure S5~S7**), which is slightly modified according to our previous work 35. EGG is not a natural ligand of GGT, and theoretically has a weak response to GGT. However, PEG is not a ligand of GGT and theoretically does not respond to GGT. The three conjugates were dispersed in the PBS buffer. Among these, GSHPD and EGGPD were in the form of zwitterionic, with a weak charge negativity. The particle sizes of conjuagtes were about 20 nm, and the charges were -5.9 ± 2.6 mV for GSHPD, -7.1 ± 3.3 mV for EGGPD, -12.5 ± 3.8 mV for PEGPD, respectively. The TEM results showed that GSHPD was well distributed without agglomeration and its particle size was 18.7 ± 2.2 nm (with a polydispersity index of 0.2) as determined using dynamic light scattering (DLS) (**Figure 3B**). We then examined the hydrolysis of GSH and EGG monomers at 10 U/mL of GGT to evaluate the GGT responsiveness of conjugates (**Figure S8**). About 45% of GSH was degraded by the GGT enzyme after 4 h of GGT enzyme degradation. However, the changes in methylene proton hydrogen detected using ^1^H-NMR showed that EGG was not degraded by enzymes, suggesting that GSH hydrolysis triggered by GGT demonstrated high specificity. Meanwhile, GSH was hydrolyzed to produce the primary amine. Therefore, the surface charge of the conjugate was monitored to verify the GGT response. The surface charge of GSHPD changed from -5.9 mV to +11 mV within 4 h, whereas the surface charge of EGGPD and PEGPD did not change significantly after incubation with 10 U/mL of GGT at pH 7.4 (**Figure 3C**). GSH-modified dendrimers exhibited faster and more specific GGT-triggered charge reversal characteristics. This efficient and specific GGT-responsive charge reversal would lead to more effective cationization-induced transcytosis in kidney tissues. The prodrug activation and releasing were then characterized using high performance liquid chromatography (**Figure S9**). When incubated with esterase (200 U/mL, dissolved in the 100 mM, pH 7.4 Tris-HCl buffer), TP released over 70% from GSHPD within 120 min whereas only released less than 5% in rat blood plasma, suggesting GSHPD could achieve intracellular drug activation and avoid premature release in the blood circulation.

We further investigated the stability, hemolysis assay, and* in vitro* cytotoxicity of GSHPD. The stability of GSHPD was evaluated in the cell culture medium after storage in different times of 1-day, 7-day,14-day, 21-day, 28-day at 25 °C in the dark condition (**Figure S10**). The particle size of GSHPD barely changed, and only 0.06% of the TP was hydrolyzed from GSHPD after four weeks storage, indicating the high stability of GSHPD. In addition, the hemolysis analysis of GSHPD in red blood cells was tested (**Figure S11**). Incubation of erythrocytes with even a higher concentration of GSHPD (10 mg/mL) resulted in a negligible hemolytic activity (<10%). The toxicity assay of GSHPD was evaluated in MPC5 and MRGEC cells using Cell Counting Kit-8 (**Figure S12**). Within the dose of 1 mg/mL, the GSHPD has no significant cytotoxicity in both MPC5 and MRGEC cells, and the cells could maintain above 90% viability. These data collectively indicated the better biocompatibility of GSHPD, providing a potential carrier for kidney-targeted drug delivery system.

### Cellular uptake of conjugates

We then screened and evaluated the expression of GGT in some renal cells (e.g., MPC5 cells, MRGEC, MRMC) or normal cell (e.g., NIH/3T3) (**Figure 3D**). The results showed that GGT was expressed in MPC5 cells, MRGECs, and MRMCs to varying degrees; however, the expression was weak in the normal cell line NIH/3T3. This result once again verified the high expression of GGT in kidney tissues, suggesting that GSHPD could enter kidney tissues through GGT-triggered transcytosis, but rarely or not into other normal tissues. The cells were then incubated with Cy5-labeled conjugates, and the uptake ability of MPC5 cells was quantitatively analyzed using flow cytometry. As shown in **Figure 3E**, GSHPD can be quickly internalized into MPC5 cells, which showed significantly higher cellular uptake efficiency than these of EGGPD (*P=0.038*) and PEGPD (*P=0.007*) after 1 h incubation. The fluorescence intensity of GSHPD reached a plateau within 1.5 h, whereas the complete absorption of PEGPD and EGGPD took 4 h, suggesting that the GSH-modified conjugate had excellent cellular uptake characteristics. Meanwhile, the pretreatment of MPC5 cells with GGsTOP, a GGT inhibitor 35, showed that the GSHPD uptake by MPC5 cells significantly reduced whereas PEGPD and EGGPD were still at low levels and had little effect, suggesting that the rapid cellular uptake of GSHPD was dominated by GGT-responsive cationization.

### Endocytosis pathway of conjugates

Chlorpromazine, genistein, wortmannin, cytochalasin D, and a low temperature of 4 ℃ were selected to investigate the endocytosis pathway of each conjugate 35, 51, 52. As shown in **Figure 3G**, chlorpromazine and low temperature significantly reduced the PEGPD and EGGPD uptake, indicating that PEGPD and EGGPD were endocytotic pathways mediated by energy-dependent and clathrin-mediated endocytosis. Another clathrin inhibitor of ikarugamycin (5 μg/mL) and ATP inhibitor of oligomycin-A (1 μg/mL) were selected to further investigate the endocytosis pathway of EGGPD and PEGPD (**Figure S13**). After pretreatment with ikarugamycin or oligomycin-A, the cellular fluorescence intensity was significantly decreased; the inhibition rates were 61.8% and 46.9% for PEGPD, and 44.1% and 56.5% for EGGPD by ikarugamycin and oligomycin-A respectively, proving the energy-dependent and clathrin-mediated endocytosis was the main endocytotic pathway for PEGPD and EGGPD. On the contrary, chlorpromazine, wortmannin, and cytochalasin D did not significantly decrease GSHPD uptake. Moreover, GSHPD uptake was significantly reduced (the inhibition rate was as high as 50%) after genistein and low-temperature pretreatment. These results suggested that the internalization of GSHPD was mediated by energy-dependent and aveolae-mediated endocytosis. GSHPD underwent rapid GGT-triggered cationization (**Figure 3C**) and rapid cell internalization (**Figure 3E**), indicating that the GGT-triggered cationization-initiated aveolae-mediated endocytosis was the main cellular uptake pathway of GSHPD.

### Vascular endothelial cell transport of conjugates

We designed a multicellular co-incubation model in this study to investigate the transport characteristics of conjugates across vascular EC. As shown in **Figure 3H**, MRGEC and MPC5 cells were inoculated into the upper and lower chambers of the Transwell plate. When the transmembrane electrical resistance of the upper chamber was greater than 800 Ω·cm^2^, the vascular endothelium was formed 53, 54 and the experiment was carried out. The conjugate was added to the upper chamber and incubated for 2 h. The upper chamber was removed, and the MPC5 cells in the lower chamber were collected to determine conjugate uptake. The cellular uptake of GSHPD was 2.1 times higher than that of PEGPD and 1.4 times higher than that of EGGPD. After pretreatment of MRGECs with GGsTOP (a GGT inhibitor) and Exo1 (an exocytosis inhibitor), GSHPD transport significantly reduced, almost at the same level as PEGPD and EGGPD uptake (**Figure 3I**). These results suggested that GGT-triggered cationization-initiated endocytosis and exocytosis were the main cellular uptake and transport pathway of GSHPD to cross the vascular endothelium.

### Subcellular distribution of conjugates

We observed the subcellular distribution of GSHPD in MPC5 cells after 2 h of incubation using CLSM. As shown in** Figure 3J,** GSHPD (red fluorescence) was absorbed into the cells and distributed in the cytoplasm but was not captured by the lysosomes (green fluorescence), indicating that GSHPD had good escape characteristics from the lysosome and could effectively protect drugs from degradation and destruction by the lysosome. On the contrary, most of PEGPD was distributed into lysosomes, and a part of EGGPD was distributed into both lysosome and cytoplasm, indicating EGGPD had to a degree the ability of lysosomal esacpe but weaker than that of GSHPD (**Figure S14**). Endocytosis was mediated by the aveolae, and GSHPD could be distributed in the Golgi apparatus or endoplasmic reticulum for reprocessing before retransport 32, 33, 35. Clathrin-mediated endocytosis can lead to the distribution of nanparticles in the lysosomes for degradation 55, 56. Therefore, the distribution of GSHPD in the cytoplasm once again proved that the GSHPD endocytosis pathway mainly depended on the aveolae-mediated endocytosis.

### Effects of conjugates on ZO1 expression in podocyte injury model

The ZO1 tight junction protein maintained podocyte structure by connecting the slit diaphragm proteins to the actin cytoskeleton. The reduction of ZO1 played a major role in podocyte injury or dysfunction, which led to proteinuria and glomerulosclerosis of childhood NS 25. We further used a puromycin aminonucleoside (PAN)-induced podocyte injury model 15, 57 to explore the pharmacological action of conjugates on ZO1 expression (**Figure S15**). Compared with the normal control (NC), the ZO1 expression was sharply reduced in PAN-induced podocyte injury model. After treatment with TP or conjugates, the reduction of ZO1 was alleviated in varying degrees. Moreover, GSHPD exhibited potent ability to upregulate the ZO1 expression in podocyte injury model, which was significantly higher than those of TP, PEGPD and EGGPD, and even recovered to a same level as that of NC. These results indicated that GSHPD conjugate might upregulate the expression of ZO1 tight junction protein to recover podocyte function and prevent renal deterioration in childhood NS.

### Blood clearance of conjugates

**Figure 4:**
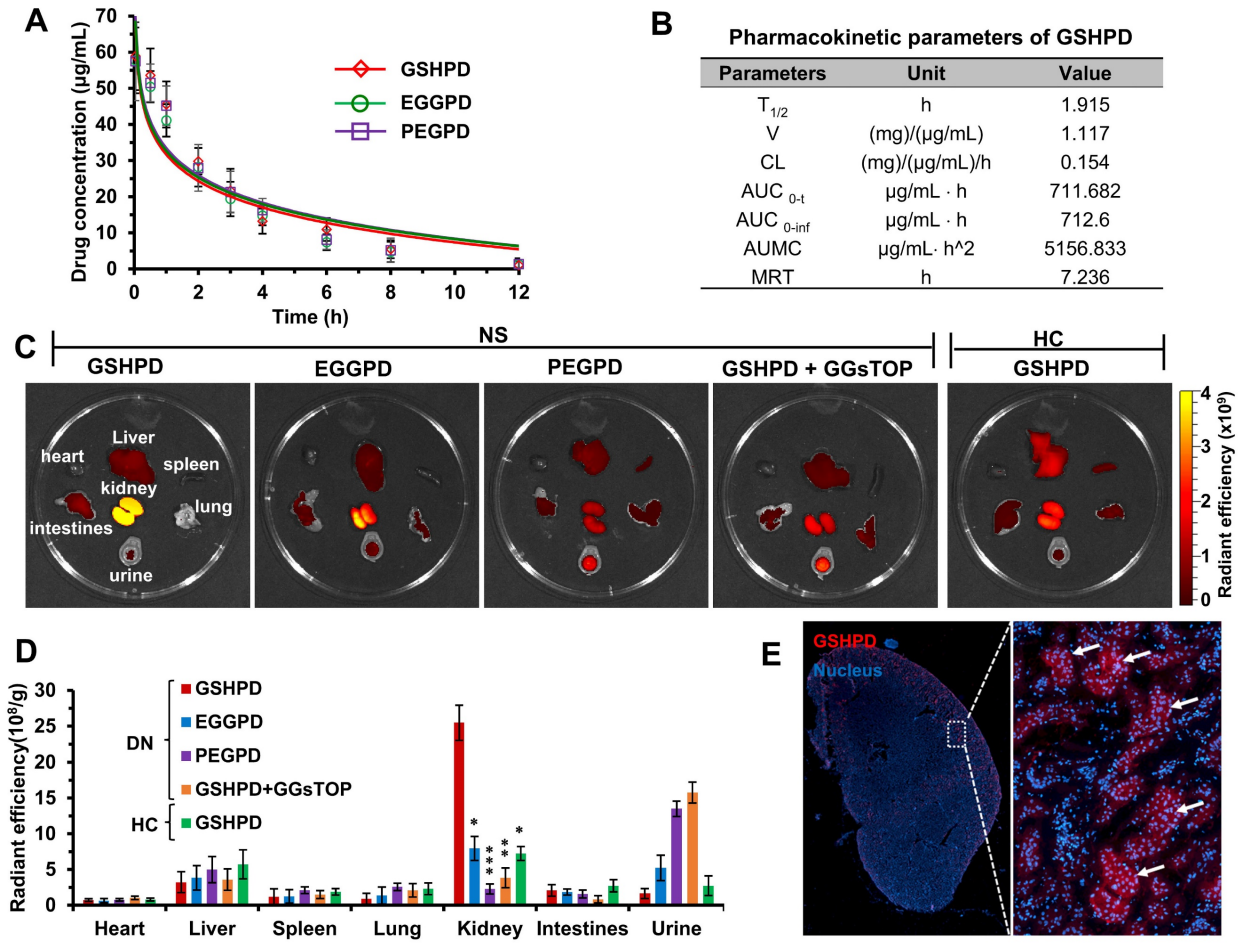
** Blood clearance, biodistribution, and renal distribution of conjugates in the ADR-induced NS juvenile rat model.** (A) Blood clearance of the conjugates. Rats were injected with GSHPD, EGGPD, and PEGPD conjugates (200 μL, equivalent to TP dose 5 mg/kg). (B) Pharmacokinetic parameters of GSHPD. *T*_2/1_: elimination half-time; *V*: apparent volume of distribution; CL: clearance ratio; AUC_0-t_: area under the curve from 0 to 12 h; AUC_0-inf_: predicted area under the curve; AUMC: area under the first moment of the plasma concentration-time curve; and MRT: mean residence time. (C) Biodistribution of the conjugates in terms of fluorescent imaging in the dissected tissues. Images (heart, liver, spleen, lung, kidney, intestines, and urine) were obtained at 24 h after injection using the IVIS Spectrum system. Additionally, GGsTOP (1 mg/kg) was injected beforehand for 2 h to investigate the inhibitory effects of the GGT inhibitor GGsTOP on biodistribution. (D) Fluorescence intensity of renal accumulation was quantitatively analyzed using Living Image 4.5 software. (E) Renal distribution of conjugates in frozen sections of tissues. Kidney tissues were excised, frozen in Tissue-Tek OCT compound, sectioned into 10-µm-thick slices, and imaged using CLSM. Compared with the GSHPD group: ^*^P < 0.05, ^**^P < 0.01, ^***^P < 0.001.

In the pre-experiment, we found that the drug concentration in blood and tissues could not be detected when juvenile rats with NS were intravenously injected with a lower dose of conjugate (equivalent to the TP dose 0.5 mg/kg). Therefore, a high equivalent dose of TP (5 mg/kg) was used in the blood clearance experiment. As shown in **Figure 4A**, the blood clearance trends of PEGPD, EGGPD, and GSHPD were basically the same. Also, the coincidence degree of the fitting curve was extremely high with no significant difference. According to the pharmacokinetic parameters of GSHPD (**Figure 4B**), its clearance half-life (*T*_2/1_) was about 1.9 h, the area under the cumulative curve was about 710 μg/mL · h, and the mean residence time (MRT) was as high as 7.2 h, which was significantly higher than that of TP reported in previous studies 58, 59, providing sufficient blood circulation time for the distribution of the kidney.

### Tissue distribution of conjugates

The tissue distribution of conjugates in juvenile rats with NS was observed using a small-animal* in vivo* imager. Given the large body size of the rats and the shielding effect of hair on fluorescence, juvenile rats with NS were killed after 24 h and the dissected organs and tissues were imaged with fluorescence. As shown in **Figure 4C-D**, GSHPD had an obvious distribution in kidney tissue from juvenile rats with NS, which was 7 times that in the liver and 2 ~ 6 times that in PEGPD and EGGPD kidneys. Meanwhile, the content of GSHPD in the urine of rats with NS was low, proving that GSHPD has significant renal targeting characteristics and good renal retention characteristics. The lower distribution of TP in the liver helped reduce the hepatotoxicity of GSHPD. Furthermore, when juvenile rats with NS were pretreated with GGsTOP (1 mg/kg) for 2 h and then injected with GSHPD, the distribution and content of GSHPD in the kidney significantly reduced and a large amount was excreted in urine, similar to PEGPD. Compared with juvenile rats with NS, when GSHPD was injected into healthy juvenile rats, the distribution of GSHPD in the kidney was significantly reduced, which was basically similar to that in the liver and less in the urine. These data suggested that the high expression of GGT in the kidney tissue of juvenile rats with NS was the critical target to mediate the renal-targeted distribution of GSHPD, and the conjugate modified by GSH was a successfully established renal-targeted drug delivery system. The kidney tissues of juvenile rats with NS injected with GSHPD were then embedded in Tissue-Tek OCT and frozen sections were made. The nuclei were stained, and the specific distribution of GSHPD in the kidney was observed using CLSM. As shown in** Figure 4E**, GSHPD was distributed mainly in the glomerulus of the renal cortex, indicating that GSHPD could be absorbed by a variety of cells in the glomerulus, such as vascular EC, podocytes, and MC, and might play a role in multicellular therapy.

### Pharmacodynamic effects of conjugates on ADR-induced NS model of juvenile rats

**Figure 5:**
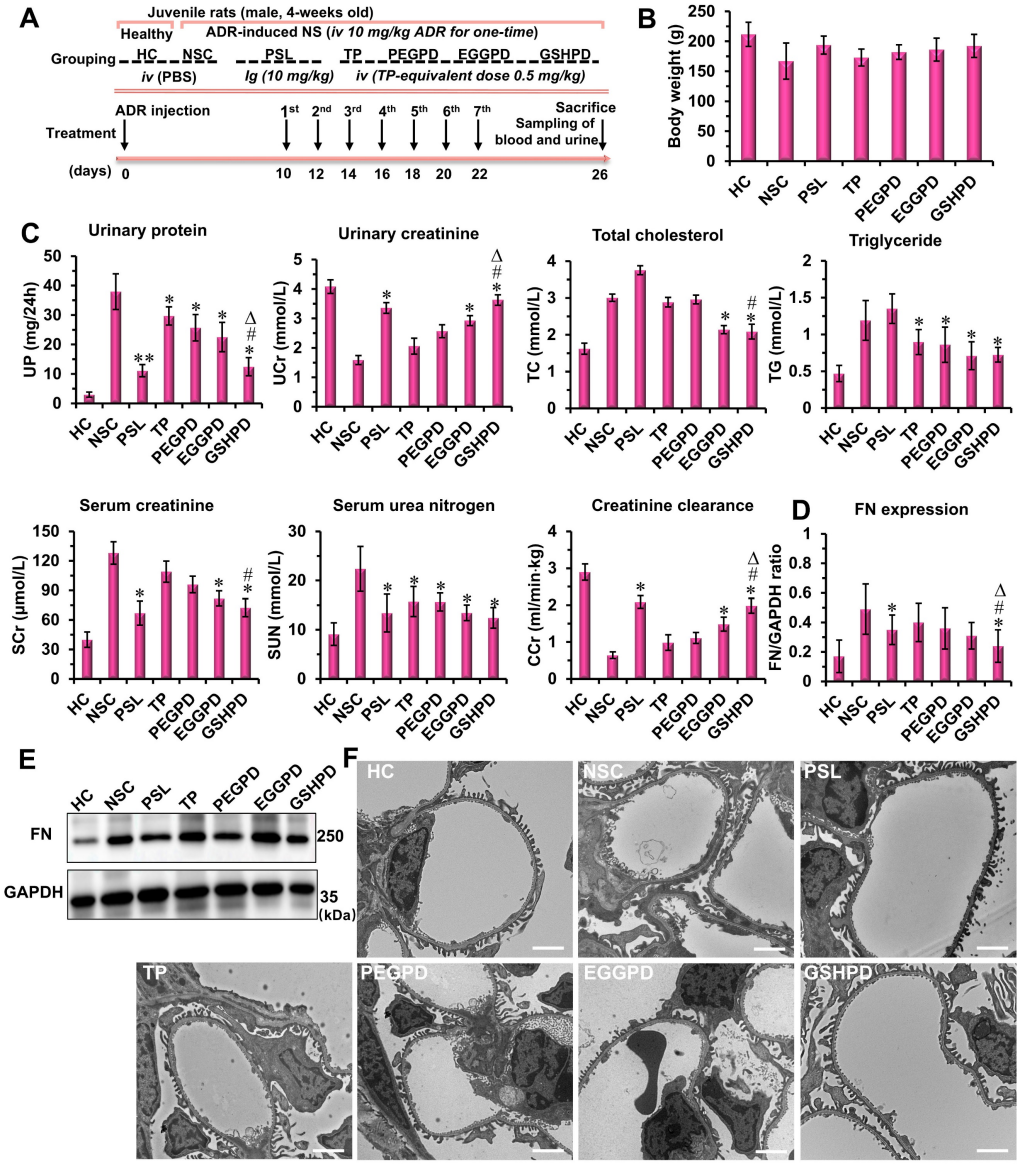
** Therapeutic efficacy of conjugates in the ADR-induced NS model of juvenile rats.** (A) Experimental design and treatment schedule. (B) Body weight of the rats at the end of the experiment. (C) Biochemical parameters in the blood and urine of rats. At the end of treatment, urinary creatinine (UCr), urinary protein (UP), total cholesterol (TC), triglyceride (TG), serum urea nitrogen (SUN), and serum creatinine (SCr) levels and creatinine clearance rate (CCr) of rats in each group were measured and analyzed. (D and E) Expression of fibronectin (FN) was evaluated using Western blot assay. The anti-fibronectin antibody (ab2413) was purchased from Abcam company and used following the manufacturer's protocols. (F) Ultrastructure of the glomerulus was visualized and analyzed using a transmission electron microscope (Scale bar = 2 μm). Compared with the NSC group: ^*^P < 0.05, ^**^P < 0.01; compared with the TP group: ^#^*P* < 0.05; compared with the PEGPD group: ^Δ^*P* < 0.05.

The pharmacodynamic effects of conjugates on juvenile rats with NS were evaluated according to the experimental design scheme shown in **Figure 5A**. The rats in the positive control group were given a prednisolone acetate solution (PSL, 10 mg/kg) by intragastric administration 7, 60, whereas the rats in the treatment groups of TP, PEGPD, EGGPD, and GSHPD conjugates were administered by tail vein injection (equivalent to TP dose of 0.5 mg/kg). Treatment was started 10 days after modeling and administered once every other day for seven consecutive times. After administration, the blood and urine samples were collected to test UP, UCr, serum TC, serum triglyceride (TG), SUN, and serum creatinine (SCr) levels and creatinine clearance rate (CCr). At the end of the treatment, the body weight of the juvenile rats was lower in the model control group (NSC) and TP group (**Figure 5B**). In contrast, no significant differences in body weight were observed among the other treatment groups. As shown in **Figure 5C**, PSL and GSHPD could significantly improve all the blood and urine indicators of rats with NS, whereas TP, PEGTP, and EGGPD could only improve some indicators of blood and urine compared with those in the NSC group, suggesting remarkable effects of PSL and GSHPD in treating NS. However, the PSL aggravated the course of the disease in regulating lipid metabolism. Also, GSHPD had a good regulatory effect on TC and TG levels, suggesting that GSHPD was better than PSL. The levels of UP, UCr, SUN, SCr, and CCr in the TP treatment group were 29.7 ± 3.08 mg/24 h, 2.06 ± 0.27 mmol/L, 15.75 ± 3.05 mmol/L, 109 ± 10.7 μmol/L, and 0.98 ±0.21 mL/(min · kg), respectively. However, the levels of UP, UCr, SUN, SCr, and CCr in the GSHPD group were 12.44 ± 10 mg/24 h, 3.63 ± 0.28 mmol/L, 12.44 ± 2.12 mmol/L, 72.45 ± 9.17 μmol/L, and 1.98 ± 0.19 mL/(min · kg), respectively. Therefore, GSHPD could significantly improve the therapeutic effect on NS compared with that in the TP group. Compared with PEGTP and EGGPD, GSHPD was advantageous in regulating most blood and urine detection indicators. These results proved a remarkable therapeutic effect of the GGT-mediated kidney-targeted drug delivery system. The rats' blood tests of alanine aminotransferase (ALT) and aspartate aminotransferase (AST) were further performed to investigate the hepatic toxicity of the conjugates (**Table S1**). The TP-treated rats showed abnormal increased levels of ALT and AST, which were about 2 times higher than that in HC group, indicating the potential toxicity to liver. In contrast, after treatment with the EGGPD and GSHPD conjugates, the levels of ALT and AST mildly increased but kept within the permission scope in clinic. Moreover, the H&E staining of major organs (e.g., liver, spleen, lung, and intestine) exhibited no significant pathological variations or adverse reactions in the EGGPD and GSHPD-treated rats, but the adverse reaction of drug-induced hepatic fibrosis has obviously happened in the TP-treated rats (**Figure S16**), proving the good safety and biocompatibility of EGGPD and GSHPD conjugates.

Fibronectin (FN) is a component of the glomerular basement membrane and mesangial matrix. It is an important extracellular matrix protein and an indicator protein in the deterioration of NS 20, 61. Western blot assay was used to detect and quantitatively analyze the expression of FN in renal tissues (**Figure 5 D and E**). PSL and GSHPD significantly reduced FN secretion and expression in kidney tissues compared with those in the NSC group. Compared with TP, GSHPD significantly improved the therapeutic effect on NS. Podocyte injury is an important factor in NS occurrence and development 62-64. Therefore, the glomerular ultrastructure and podocyte changes were observed and analyzed using TEM. As shown in **Figure 5F**, podocytes had regular podocyte-like structures and the basement membrane was thin in the HC group. However, podocytes were severely damaged, the podocyte-like structures showed irregular disappearance or adhesion, the basement membrane was significantly thickened, and the matrix accumulation increased in the NSC group. After treatment with PSL and conjugates, the degree of podocyte damage improved to varying degrees, and GSHPD showed a more obvious improvement. Meanwhile, podocytes restored the regular podocyte-like structure, the basement membrane became thinner, and the membrane pores recovered after GSHPD treatment.

## Conclusions

In summary, we found GGT was highly expressed in juvenile rats with NS and mainly distributed in the renal capillary endothelial cells and podocytes. We then designed and constructed a GSH-modified TP-conjugated polyamide amine dendrimer (GSHPD) using GGT as the targeted receptor of the kidney-targeted drug delivery system for childhood NS therapy. GSHPD was composed of G5-generation dendrimer PAMAM chemically coupled with TP prodrug and GSH. GSHPD was circulated to the renal capillary network of NS through stable blood circulation and was cationized by GGT on the surface of endothelial cells to enhance renal drug distribution through the cationization-initiated aveolae-mediated transcytosis. The cationic GSHPD diffused and infiltrated into the kidney tissue to a variety of cells through continuous transcytosis to achieve efficient cellular uptake. The TP prodrug released the active TP under the catalysis of renal cell carboxylesterase. GSHPD significantly improved the therapeutic effect of TP on juvenile rats with NS, improved renal physiological parameters, reduced the expression of renal fibronectin, and restored podocyte function. This GGT-responsive kidney-targeted drug delivery system can overcome the renal physiological barrier and cellular uptake barrier by cationization-initiated transcytosis, and achieve effective treatment of juvenile rats with NS, providing a potential strategy for treating renal diseases in children.

## Methods

### Synthesis and characterization of conjugates

The synthetic route of conjugates was followed using GSHPD as an example (**Figure S2**) according to our previous work 35, 65. First, the TP prodrug was synthesized. 6-Acrylate hydroxyhexyl acetate (1.1 g, 6.0 mmol) and dimethylaminopyridine (2.2 g, 18.0 mmol) were dissolved in 50 mL of dry-dehydrated dichloromethane protected under argon atmosphere. Triphosgene (0.6 g, 2.0 mmol) was then added and stirred at room temperature for 30 min. TP (2.1 g, 6.0 mmol, dissolved in 30 mL of dichloromethane) was added dropwise through a constant-pressure funnel, and the reaction mixture was stirred for 8 h. After filtering and removing the insoluble matter and evaporating all solvents, the residue was diluted with ethyl acetate, washed with deionized water three times, and washed with sodium chloride two times. The organic layer was dried, filtered using anhydrous MgSO_4_, and concentrated in a rotary evaporator. The crude product was purified by column chromatography with ethyl acetate as an eluent (100% ethyl acetate), and the white solid powder TP prodrug (1.5 g, yield about 47%) was obtained. The characteristics of ^1^H nuclear magnetic resonance spectroscopy (^1^H-NMR, 400 MHz, CDCl_3_) were as follows: δ 6.38 (s, 1.1H), 6.10 (s, 1.0H), 5.59 (s, 1.1H), 4.91 (s, 2.1H), 4.47 (s, 1.0H), 4.0 (s, 4.0H), 3.18 (s, 2.0H), 2.62-2.26 (D, 15.3H), 1.66 (s, 3.0H), and 0.91 (s, 3.0H). The molecular weight of TP prodrug was determined to be 576.28 in the form of [M+NH_4_]^+^ according to mass spectrometry (**Figure S3**). TP prodrug (71 mg, 130 µmol) and PAMAM (288.3 mg, equivalent to 1.3 mmol of active NH_2_ fraction) were dissolved in 20 mL of DMF and stirred at 45℃ for 12 h. The remaining TP prodrug in the reaction was monitored using high-performance liquid chromatography (HPLC). When the content of the TP prodrug in the reaction solution was less than 5%, dialysis purification (the cutoff molecular weight of the dialysis bag = 10 kDa) was performed in deionized water (the volume ratio of the sample to the dialysis buffer was 1:100), and the dialysis solution was changed every 6 h for 24 h. After freeze-drying, the PAMAM-TP conjugate (about 320 mg, yield 85%) was obtained, and confirmed by the ^1^H-NMR (**Figure S4**). The HPLC results revealed that the coupling rate was about 10%, and each PAMAM had 12 TP prodrug couplings. Subsequently, Boc-γ-glu (otbu)-Cys (Trt)-Gly (176.3 mg, 250 µmol), PAMAM-TP conjugate (30 mg, equivalent to 118 µmol of active NH_2_ fraction), PyBop (260 mg, 0.5 mmol), HoBt (68 mg, 0.5 mmol), and 80 μL of DIPEA were dissolved in 5 mL of DMF and stirred at room temperature for 12 h. The solution was first dialyzed in DMF for 12 h, and the fresh dialysate was replaced every 3 h at 4 ℃. The solution was dropped into 10 times the volume of DCM/TFA (*v*/*v*, 1/1) mixed solution and stirred for 2 h. Then, the solution was removed by vacuum pumping on the rotary evaporator. The light-yellow solid of GSHPD (60 mg, yield about 80%) was obtained after freeze-drying and precipitation with frozen ether twice. The structure characteristic of GSHPD was proved by ^1^H-NMR (**Figure S5**). GSHPD was stored at -20 ℃ and dissolved in deionized water or PBS solution when used. Meanwhile, two control conjugates were synthesized: GGT weakly responsive EGGPD and GGT unresponsive PEGPD. EGGPD (**Figure S6**) was synthesized using Boc-γ-glu (otbu)-Gly-Gly in the same way. PEG succinyl propionate (100 mg, 180 µmol) and PAMAM-TP conjugate (30 mg, equivalent to 118 µmol of active NH_2_) were dissolved in 10 mL of DMF and stirred at room temperature for 12 h. The solution was dialyzed in deionized water for 12 h, and the fresh dialysate was changed every 3 h at 4 ℃. After freeze-drying, PEGPD (about 80 mg, yield about 90%) was obtained (**Figure S7**). Meanwhile, the Cy5-labeled PAMAM was synthesized using PAMAM and the active ester of Cy5-NHS *via* amidation reaction, in which a PAMAM conjugate coupling with two Cy5 fluorescent molecules. The Cy5-labeled conjugates of GSHPD, EGGPD, and PEGPD were then synthesized using the Cy5-labeled PAMAM according to the above-described protocol. The Cy5-labeled conjugates were used for *in vivo* and* in vitro* imaging experiments.

### Characterization analysis of the pharmaceutical properties of conjugates

A total of 100 μL conjugate was diluted with 900 μL of PBS solution and placed in a pool to measure the zeta potential. Each sample was measured three times, and the average value was taken. Then, 20 μL of the conjugate was dropped onto a 100-mesh copper mesh, allowed to stand for 1 min, and then negatively stained with uranyl acetate solution for 20 s. Subsequently, the solvent was evaporated under an infrared baking lamp, and the morphology and structure of the conjugates were observed using a transmission electron microscope (TEM). GGT (10 U/mL) was added to the conjugate for co-incubation with shaking at 100 rpm and 37 ℃. After incubation for some time, each sample (100 μL) was transferred to 900 μL of PBS solution and placed in a potential pool, and the zeta potential was measured.

### Esterase-responsive drug release

GSHPD (equivalent to TP dose of 0.5 mg/mL) were respectively dissolved and incubated in the rat blood plasma (total protein concentration about 60 mg/mL) and porcine liver esterase (200 U/mL, dissolved in the 100 mM, pH 7.4 Tris-HCl buffer) at 37 °C. At timed intervals, 100 μL of the solution was sampled and subjected to HPLC. The content of TP were determined by HPLC, using the following parameters: chromatographic column [Platisil ODS type (150 mm × 4.6 mm, 5 μm], mobile phase (acetonitrile-0.02 mol/L sodium dihydrogen phosphate aqueous solution), flow rate (1.0 mL/min), column temperature (25 ℃), and detection wavelength (210 nm). This was followed by specificity investigation, standard curve preparation, and stability test. The esterase-pretreated plasma or tissue samples of TP *in vivo* was accomplished as follows: 100 μL of the supernatant of plasma or tissue homogenate was added to 400 μL of methanol, shaken for 30 s, and centrifuged at 8000 rpm for 10 min. Then, 300 μL of the supernatant was taken and volatilized with nitrogen at 35 ℃. The residue was redissolved in 150 μL of methanol/acetonitrile, shaken for 30 s, and ultrasonicated for 2 min. Finally, the conjugates treated earlier were filtered through a 0.22-μm microporous membrane, followed by HPLC sample analysis.

### Cellular uptake assay

The logarithmic-phase MPC5 cells or MRGEC were inoculated into 12-well plates. Once the cells adhered completely to the wall, a certain concentration of fluorescently labeled conjugate was added. The medium was poured after 15 min, 30 min, 1 h, 1.5 h, 2 h, 3 h, and 4 h of culture, washed with PBS solution, digested with 0.25% trypsin solution (containing 0.02% EDTA), collected in the flow tube, centrifuged, and washed with PBS solution once. Finally, the cells were dispersed, suspended in 0.5 mL PBS solution, and detected using flow cytometry. The excitation and emission wavelengths of Cy5 were set at 640 and 662-737 nm, respectively. The fluorescence intensity absorbed into the cells was quantitatively analyzed, and each sample was processed three times.

### Endocytosis assay

The logarithmic MPC5 cells or MRGECs were seeded in 12-well plates. After 12 h of culture, the medium in each well was replaced with 2 mL of serum-free medium. After 10 min of stabilization, chlorpromazine (50 µm, an inhibitor of clathrin-mediated endocytosis), genistein (7.5 µm, an inhibitor of caveolae-mediated endocytosis), wortmannin (5 µm, an inhibitor of macropinocytosis), and cytochalasin D (5 µm, bound to the growth ends of actin nuclei and F-actin, inhibiting polymerization and inducing actin depolymerization) were added 66. After incubating at 4 ℃ for 1 h, fluorescent conjugates were added to cells and cultured for 2 h. The culture medium was then removed, the cells were washed with 2 mg/mL of heparin sodium solution once, digested with 0.25% trypsin (containing 0.02% EDTA), and collected in a flow tube, followed by centrifugation and washing with PBS once. Finally, the cells were dispersed, suspended in 0.5 mL PBS solution, and detected by flow cytometry. The excitation and emission wavelengths of Cy5 were set at 640 and 662-737 nm, respectively. The cells treated with conjugate without inhibitor served as the positive control, whereas those without any treatment served as the negative control. Each sample was processed three times.

### Transmembrane transport and transport mechanism

The logarithmic-phase MRGEC or MPC5 cells were inoculated into the upper and lower chambers of Transwell 12-well plates (Corning 3402, with membrane area of 1.12 cm^2^ and membrane pore size of 3 μm). The upper ventricle mimicked the formation of vascular endothelium. The transmembrane electrical resistance was measured every 2 days. The experiment was conducted when the transmembrane electrical resistance was greater than 800 Ω/cm^2^. The medium in each well was replaced with 5 mL of serum-free medium, and conjugates were added to the upper chamber to observe its transport. After incubating for 2 h, the upper chamber was removed. The MPC5 cells in the lower chamber were washed with 2 mg/mL heparin sodium solution once, digested with 0.25% trypsin solution (containing 0.02% EDTA), and collected in a flow tube, followed by centrifugation and washing with PBS once. Finally, the cells were dispersed, suspended in 0.5 mL of PBS solution, and detected by flow cytometry. The excitation and emission wavelengths of Cy5 were set at 640 and 662-737 nm, respectively. Meanwhile, the transport properties of the conjugates were verified using GGsTOP (a GGT inhibitor) and Exo1 (exocytosis inhibitor) 66.

### Subcellular distribution assay

The logarithmic-phase MPC5 cells were inoculated in a glass-bottom culture dish (with a radius of 15 mm) at a density of 1.0 × 10^5^ cells/well and cultured for 24 h to fully adhere to the wall of the culture dish. The culture medium in each well was replaced with 1 mL of serum-free medium and incubated with fluorescently labeled conjugates for different durations to observe the distribution of conjugates (within 2 h, performing live-cell staining and then adding conjugates). LysoTracker Green (at a concentration of 200 nM, excitation wavelength: 488 nm, emission wavelength: 511 nm) was dropped into the culture dish for 45 min. Hoechst 33342 (at a concentration of 5 μg/mL, excitation wavelength: 405 nm, emission wavelength: 461 nm) was added to the culture dish for 25 min. Finally, the culture medium was discarded, and the cells were washed once with PBS and placed under the confocal laser scanning microscope (CLSM) to observe the distribution characteristics of the intercellular conjugates.

### Establishment of the NS juvenile rat model

The NS juvenile rat model was established following the methods described in the reported studies 46, 47, with a few modifications. Briefly, 4-week-old male juvenile Sprague-Dawley (SD) rats were selected and injected with hydroxydaunorubicin (ADR) through the tail vein at a dose of 10 mg/kg once. The urine protein content was measured, and the experimental animals were selected based on the urine protein content more than 10 mg/24 h. Meanwhile, the urinary creatinine (UCr) content was measured, and a proteinuria index of more than 20 was used to determine the success of modeling. The proteinuria index was calculated using the following formula: proteinuria index = proteinuria concentration/creatinine concentration × 100%.

### Western blot assay

A total of 50 mg freshly isolated kidney tissue was added to 0.5 mL of enhanced cell lysate to lyse kidney tissues. An enhanced BCA protein detection kit was used to determine the total protein concentration, and the total protein was diluted to a concentration of 5 μg/μL. The total protein was diluted with a loading buffer, denatured at 95 ℃, and loaded onto SDS-polyacrylamide gel electrophoresis to separate the protein bands. The protein was electro-transferred to the nitrocellulose membrane and incubated using 5% skimmed milk powder by shaking at room temperature for 1 h. The nitrocellulose membrane was then incubated with the primary antibody solution [according to the target experiment, selecting the anti-GGT antibody (Cat. No. #ab55138) or the anti-fibronectin antibody (Cat. No. #ab2413) and then diluting with primary antibody dilution buffer in a ratio of 1:1000] overnight at 4 ℃. The membrane was washed with Tris-buffered saline Tween-20 (TBST) three times and incubated with horseradish peroxidase-labeled goat anti-rabbit or goat anti-mouse secondary antibody (1:1000) at room temperature for 2 h, followed by washing with TBST three times. Finally, the membrane was subjected to chemiluminescence staining, and the images were captured with a chemiluminescence imaging device.

### Immunohistochemical assay

The kidney tissues were fixed with 4% paraformaldehyde at room temperature, embedded in paraffin, and cut into sections. These sections were dehydrated in xylene, rehydrated in a series of gradient ethanol, repaired with citric acid buffer (pH = 6.0), and heated in a microwave oven for 15 min. They were treated with 3% hydrogen peroxide for 10 min at room temperature to inhibit endogenous peroxidase activity. Further, they were blocked with 5% goat serum for 30 min and then incubated with an anti-GGT antibody (Cat. No. #ab55138) at 4 ℃ overnight. After washing with PBS three times, the tissue sections were incubated with goat anti-rabbit IgG for 1 h. The sections were then washed again three times with PBS and stained with DAPI. Finally, the tissue sections were observed under the microscope and analyzed.

### Blood clearance assay

When the therapeutic dose of TP is 0.5 mg/kg, the drug concentration in blood and tissue cannot be easily detected. Therefore, this study used high-dose TP (5 mg/kg) for the blood clearance assay. The conjugates (equivalent to a TP dose of 5 mg/kg, *n* = 4) were intravenously injected into juvenile rats with NS. At different time points (2 min, 15 min, 30 min, 1 h, 2 h, 3 h, 4 h, 6 h, 8 h, and 12 h), 50 µL of the blood was collected from the rat orbital vein and mixed with 50 µL of heparin solution (1 mg/mL). At the end of the experiment, the juvenile rats were killed and blood or kidney homogenate was collected to determine the concentration of the TP prodrug. The aforementioned blood and kidney homogenates were centrifuged at 4 ℃ for 5 min (5000 rpm) [that could be incubated with 50 µL of esterase (100 U/mL) for 30 min as appropriate], and 100 µL of the supernatant was diluted with 950 µL of acetonitrile for TP release and extraction. The mixture thus obtained was subjected to vortex and ultrasonic treatment and then centrifuged at 5000 rpm for 5 min. Finally, 500 µL of the supernatant was collected and concentrated for HPLC analysis. The TP content was calculated according to the standard curve, and the pharmacokinetic parameters of TP were also analyzed.

### Tissue distribution assay

The fluorescently labeled conjugates were injected intravenously into juvenile rats with NS (equivalent to the TP dose of 5 mg/kg, *n* = 4). An *in vivo* imaging system (IVIS Lumina II, PerkinElmer) was used to quantitatively analyze the biological distribution of conjugates 24 h after injection. The urine of each rat was collected in the metabolic cage. The main organs, including the heart, liver, spleen, lung, kidney, and intestines, were collected after killing juvenile rats with NS. These organs were washed with PBS solution, weighed, and imaged. The IVIS Lumina II imaging system was used to record the total fluorescence intensity of the isolated organs and urine.

### Pharmacodynamic experiments

Thirty-six juvenile rats with NS were randomly divided into six groups: the NS model control group (NSC), the positive control group (PSL, intragastric administration of prednisolone acetate solution at a dose of 10 mg/kg), the free drug group (TP), and three conjugate groups (PEGPD, EGGPD, and GSHPD, with intravenous administration of the drug through the tail vein, equivalent to 0.5 mg/kg TP). At the same time, six healthy juvenile rats without modeling were selected as healthy control (HC). The mice in the HC and NSC groups were injected intravenously with PBS buffer. All juvenile rats were administered once every other day for seven consecutive days. At the end of the treatment, the urine of each rat was collected in a metabolic cage, and blood was collected from the heart after anesthesia. The kidney tissues were dissected and collected after death. The 24-h urinary protein (24-h UP) excretion and UCr levels were measured using an ELISA kit. The whole-blood samples were centrifuged to determine serum urea nitrogen (SUN), serum total cholesterol (TC), serum triglyceride (TG), and serum creatinine (Scr) levels and creatinine clearance rate (CCr). One kidney of each rat was fixed with 4% paraformaldehyde and embedded in paraffin. The expression of fibronectin (FN) in the kidney was detected and analyzed by immunohistochemical assay. The other kidney was isolated and fixed at 4 ℃ for 24 h in a 10-fold amount of fixed solution (pH 7.4, 3% glutaraldehyde, 75 mM sodium dimethylarsenate, 4% pyrrolidone, 0.05% CaCl_2_, and 1% sucrose). The tissue block was trimmed to 4-5 mm, immersed in 7% agarose, cut into 0.25- to 1-mm-thick slices using a tissue blade. The slices were then rinsed with 100 mM sodium dimethylarsenate buffer, fixed with 1% osmic acid solution for 2 h, rinsed with PBS solution, dyed at 37 ℃ with 2% uranium acetate for 48 h, dehydrated and dried with acetone gradient, and immersed in epoxy resin. The embedded tissue was cut into 60-80 nm sections using an ultrathin microtome, and the glomerular ultrastructure was observed using the TEM after staining with lead citrate. The CCr content was calculated using the following equation: CCr [mL/(min · kg)] = UCr × 24-h UV (SCr × body weight × 24 × 60).

### Data analysis

Unless otherwise specified, all results were subjected to at least three parallel experiments. The data were expressed as mean ± standard deviation. The selection of microscopic images conformed to the principle of randomness. The quantitative analysis of images was conducted using Living image 4.5 software, Image-Pro Plus 6.0, and ImageJ software. GraphPad Prism 8 was used for mapping and statistical analysis of data. The unpaired-sample two-tailed Student *t* test was used for analyzing continuous variables. The one-way analysis of variance followed by Tukey's correction and 95% confidence interval was used for analyzing classified variables. In all tests, a *P* value <0.05 indicated a statistically significant difference.

## Supplementary Material

Additional experimental details and data, including: Figure S1. Immunofluorescent staining of GGT receptor in the kidney tissues; Figure S2. Schematic diagram of the chemical synthesis of the GSHPD; Figure S3. The MALDI-TOF-MS spectrum of TP prodrug; Figure S4. The ^1^H NMR spectrum of PAMAM-TP. Figure S5. The ^1^H NMR spectrum of GSHPD; Figure S6. The ^1^H NMR spectrum of EGGPD; Figure S7. The ^1^H NMR spectrum of PEGPD; Figure S8. Structural changes of EGG and GSH on the ^1^H NMR spectrum; Figure S9. The percent of TP releasing from the GSHPD after incubation with rat blood plasma and porcine liver esterase; Figure S10. The stability assay of GSHPD in the cell culture medium; Figure S11. The hemolysis analysis of GSHPD in red blood cells; Figure S12. The toxicity assay of GSHPD in renal cells of MPC5 and MRGEC; Figure S13. The inhibitory effects of clathrin inhibitor and ATP inhibitor on cellular uptake of EGGPD and PEGPD; Figure S14. Subcellular distribution of PEGPD or EGGPD after 2 h incubation in MPC5 cells; Figure S15. The effects of conjugates on ZO1 expression in podocyte injury model; Figure S16. H&E staining of the major organs after treatment with the conjugates in juvenile rats with NS; Table S1. The serum biochemistry analysis of juvenile rats with NS after treatments with the congjugates.
