# Supplementary material for: Enzyme-activatable kidney-targeted dendrimer-drug conjugate for efficient childhood nephrotic syndrome therapy
Source: Theranostics. 2024 Oct 21;14(18):6991–7006. doi: 10.7150/thno.101606 (PMC11610141; doi:10.7150/thno.101606)
Supplement: Supplementary file 1 — Additional experimental details and data, including: Figure S1. Immunofluorescent staining of GGT receptor in the kidney tissues; Figure S2. Schematic diagram of the chemical synthesis of the GSHPD; Figure S3. The MALDI-TOF-MS spectrum of TP prodrug; Figure S4. The 1H NMR spectrum of PAMAM-TP. Figure S5. The 1H NMR spectrum of GSHPD; Figure S6. The 1H NMR spectrum of EGGPD; Figure S7. The 1H NMR spectrum of PEGPD; Figure S8. Structural changes of EGG and GSH on the 1H NMR spectrum; Figure S9. The percent of TP releasing from the GSHPD after incubation with rat blood plasma and porcine liver esterase; Figure S10. The stability assay of GSHPD in the cell culture medium; Figure S11. The hemolysis analysis of GSHPD in red blood cells; Figure S12. The toxicity assay of GSHPD in renal cells of MPC5 and MRGEC; Figure S13. The inhibitory effects of clathrin inhibitor and ATP inhibitor on cellular uptake of EGGPD and PEGPD; Figure S14. Subcellular distribution of PEGPD or EGGPD after 2 h incubation in MPC5 cells; Figure S15. The effects of conjugates on ZO1 expression in podocyte injury model; Figure S16. H&E staining of the major organs after treatment with the conjugates in juvenile rats with NS; Table S1. The serum biochemistry analysis of juvenile rats with NS after treatments with the congjugates. [file thnov14p6991s1.pdf]

Supporting Information

Enzyme-activatable kidney-targeted dendrimer-drug conjugate for efficient childhood nephrotic syndrome therapy

Danfei Chen,[1] Junjun Xu,[2] Sha Lv,[2] Xiaoqin Jin,[3] Yuyan Chen,[1] Haifang Cai,[1] Qili Wang,[1] Xiaobo Xuan,[1] Guowei Wang,[4,5,*] Weidong Fei,[6,*] and Jian Chen[1,*]

[1] Department of Pediatrics, The First Affiliated Hospital of Zhejiang Chinese Medical University (Zhejiang Provincial Hospital of Chinese Medicine), Zhejiang Chinese Medical University, Hangzhou 310006, China.

[2] Department of Pharmacy, The Second Affiliated Hospital of Zhejiang University School of Medicine, Zhejiang University, Hangzhou 310009, China.

[3] Academy of Chinese Medical Sciences, Zhejiang Chinese Medical University, Hangzhou 310053, China.

[4] Research Center of Ultrasound in Medicine and Biomedical Engineering, The Second Affiliated Hospital of Zhejiang University School of Medicine, Zhejiang University, Hangzhou 310009, China.

[5] ZJU-Hangzhou Global Scientific and Technological Innovation Center, Zhejiang University, Hangzhou 311215, China.

[6] Women's Hospital, Zhejiang University School of Medicine, Hangzhou 310006, China.

[*]Corresponding author, E-mail: wangguowei@zju.edu.cn, feiweidong@zju.edu.cn, and chenj670@163.com

Materials

All chemical reagents, unless otherwise specified, were purchased from Aladdin Reagent Inc. or Sigma–Aldrich. PEG succinimidyl propionate (PEG-NHS, with molecular weight (MW): 550 Da) was purchased from Shanghai Yanyi Biotechnology Co., Ltd. (Shanghai, China). The Boc-γ-glu(otbu)-Cys(Trt)-Gly (pro-GSH) and Boc-γ-glu(otbu)-Gly-Gly (pro-EGG)

were purchased from ChinaPeptides Co. Ltd. (Shanghai, China). Cy5-N-hydroxysuccinimide ester (Cy5-NHS) was purchased from Hangzhou Harong Biotech. Co., Ltd. (Hangzhou, China). The anti-fibronectin antibody was purchased from Abcam Biotech. Hoechst 33342 and LysoTracker Green DND26 were purchased from Thermo Fisher Scientific. The protein detection, urea nitrogen detection, and creatinine detection ELISA kits were purchased from Nanjing Jiancheng Bioengineering Institute (Nanjing, China). DMEM, fetal bovine serum (FBS), and 0.25% trypsin were purchased from the Gibco Biotech Company. The GGT activity detection kit (Cat. No. BC1225) was purchased from Beijing SolarBio SciTech. Co., Ltd. (Beijing, China). Chlorpromazine, genistein, wortmannin, cytochalasin D, puromycin aminonucleoside, ikarugamycin, and oligomycin-A were purchased from Med Chem Express.

Cell culture and animals

Mouse podocyte clone-5 (MPC5), mouse glomerular endothelial cells (MRGEC), mouse mesangial cells (MRMC), and mouse embryonic fibroblasts (NIH/3T3) were provided by the Institute of Biological Sciences, Chinese Academy of Sciences. The cell culture system comprised DMEM or 1640 medium containing 10% FBS, 100 U/mL penicillin, and 100 μg/mL streptomycin. The cells were cultured at 37 °C in the presence of 5% CO_2.

The juvenile male Sprague-Dawley (SD) rats (4 weeks old) were provided and raised by the Experimental Animal Center of Zhejiang Chinese Medical University. All animal experiments were approved by the Animal Ethics Committee of Zhejiang Chinese Medical University (Approval No. IACUC-20230913-02).

Figures and Tables

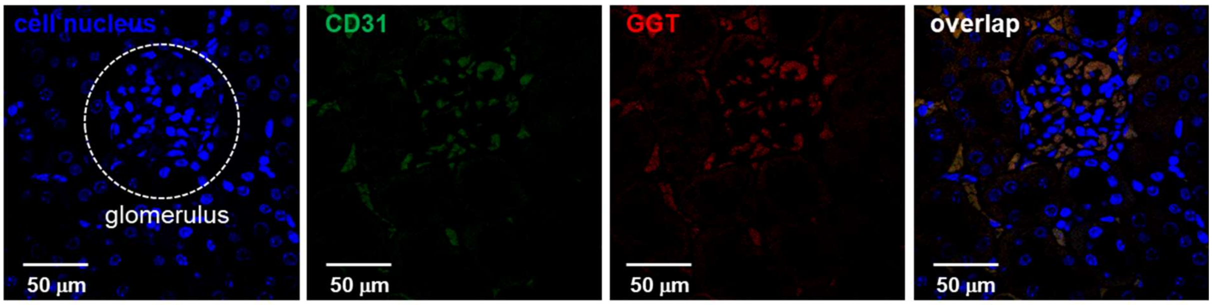

Figure S1. Immunofluorescent staining of GGT receptor in the kidney tissues. Immunofluorescence co-localization analysis was used to examine GGT expression, the green fluorescence indicates the vascular endothelial cells marker of CD31, red fluorescence

indicates GGT receptor, and blue fluorescence represents nuclei, Scale bar = 50 μm.

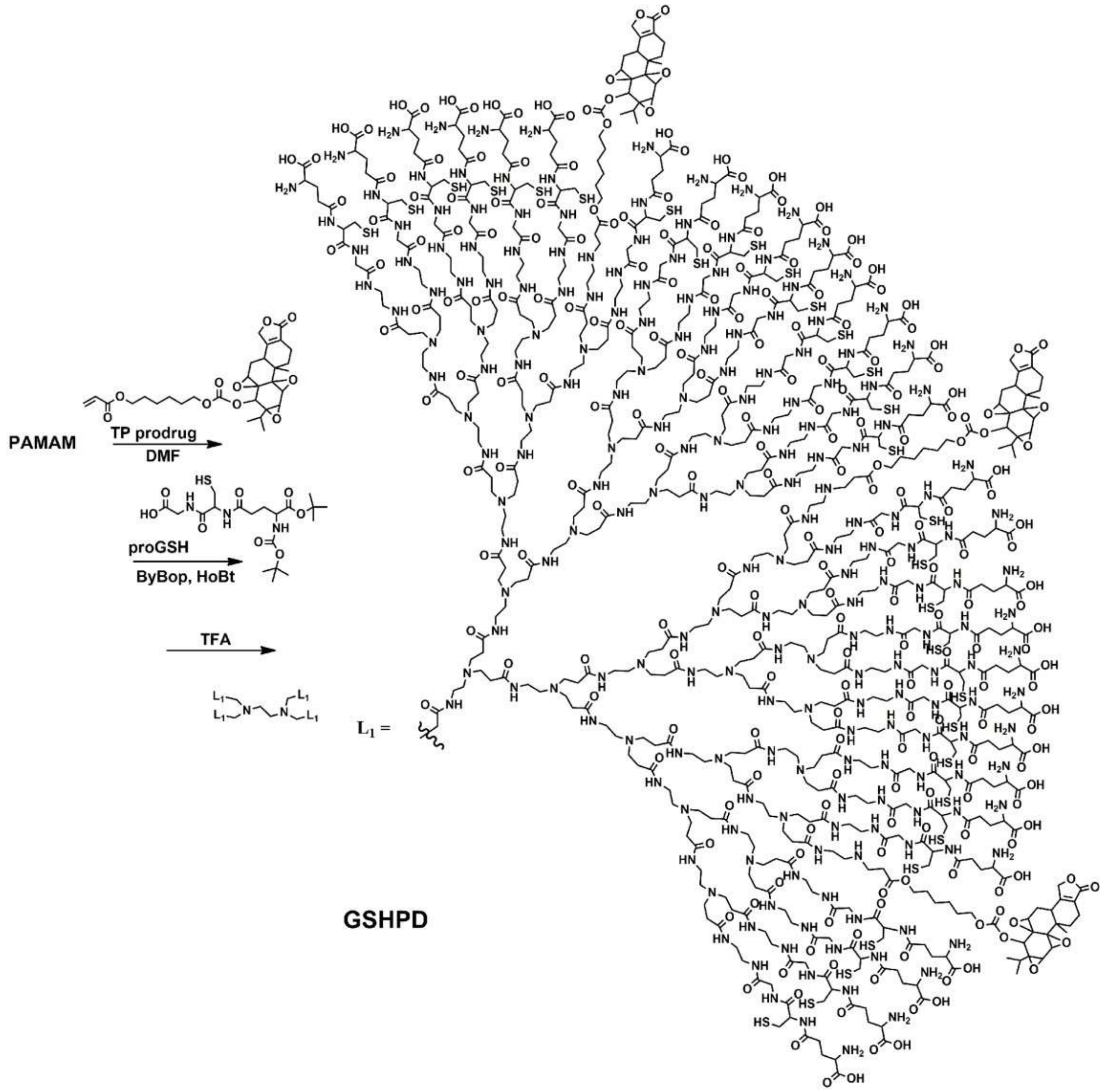

Figure S2. Schematic diagram of the chemical synthesis of the *γ*-glutamyl transpeptidase-triggered charge-switchable dendrimer-triptolide conjugate (GSHPD). The GSHPD conjugate is synthesized by conjugating triptolide and glutathione on polyamidoamine dendrimer by using the hexanediol carbonic ester linker and the GSH precursor of boc-*γ*-Glu(otbu)-Cys(Trt)-Gly.

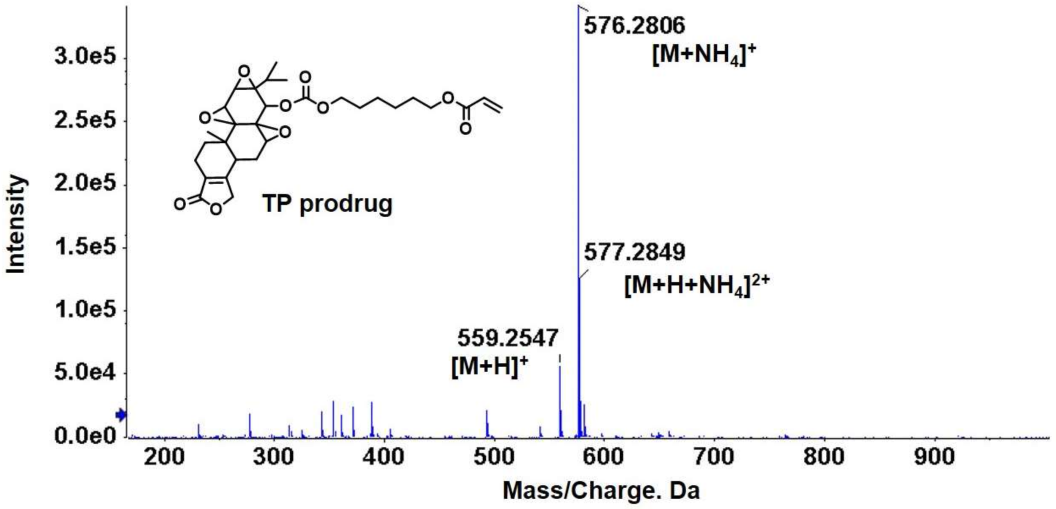

Figure S3. The MALDI-TOF-MS spectrum of TP prodrug.

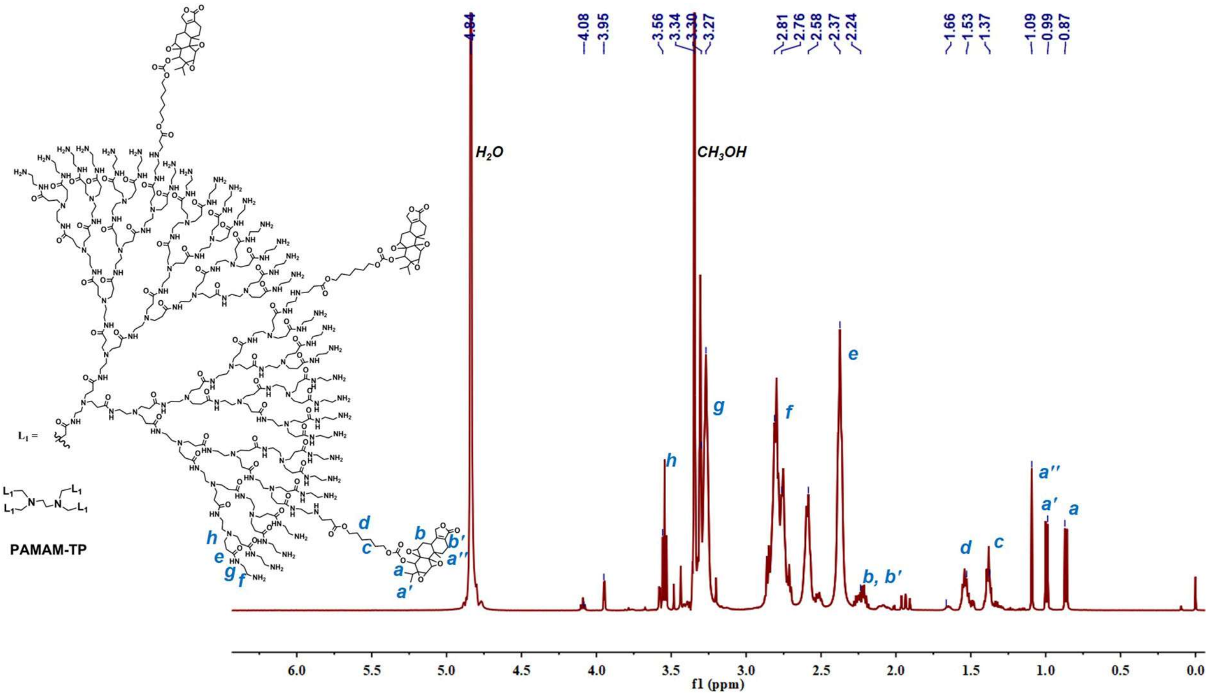

Figure S4. The ^{1}H NMR spectrum of PAMAM-TP.

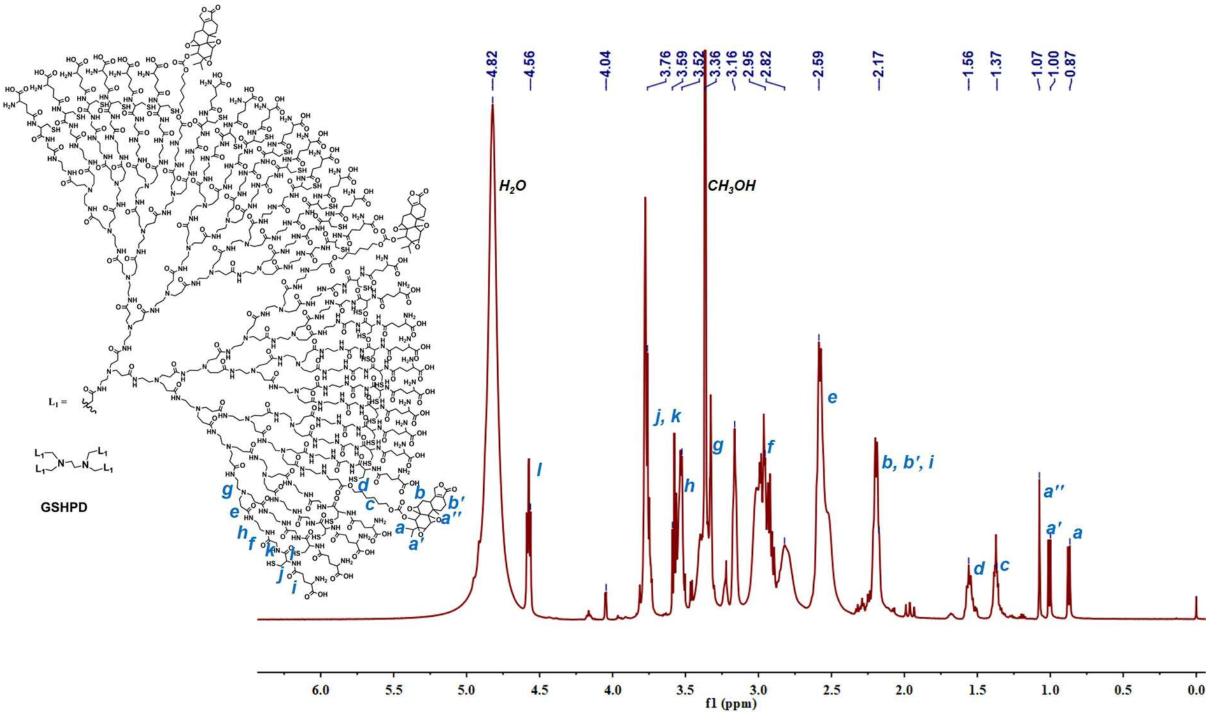

Figure S5. The ^{1}H NMR spectrum of GSHPD.

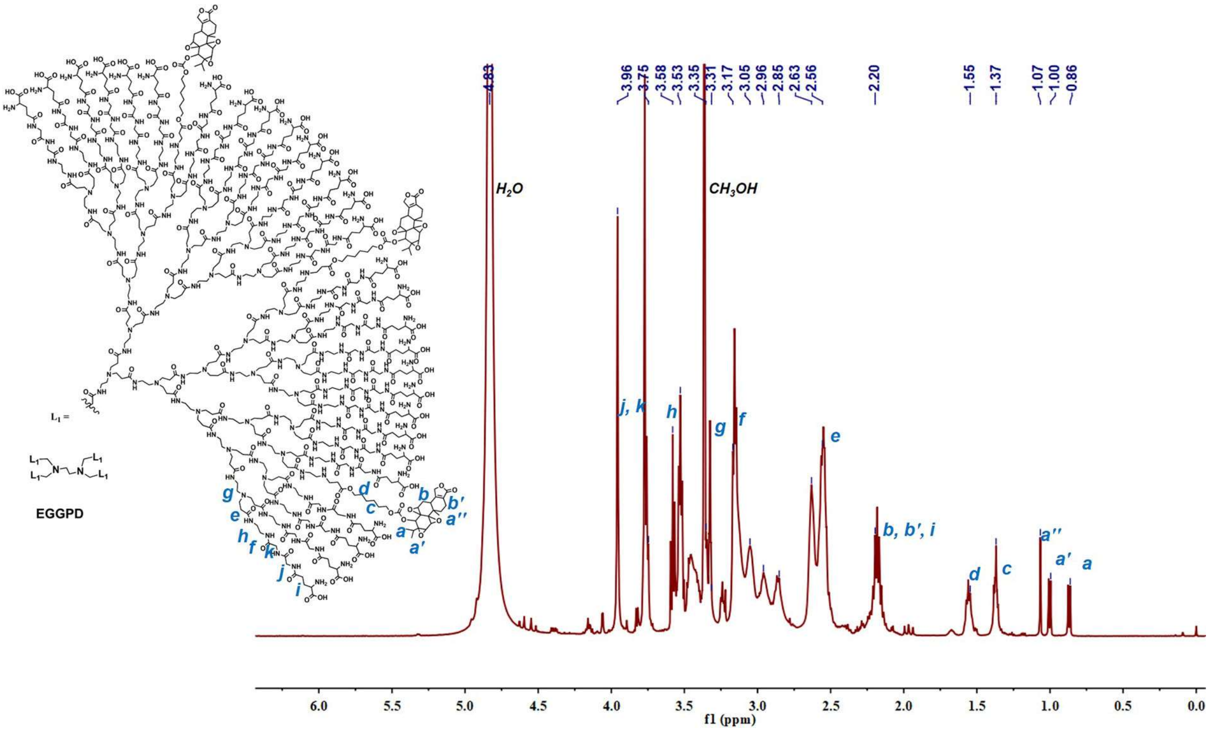

Figure S6. The ^{1}H NMR spectrum of EGGPD.

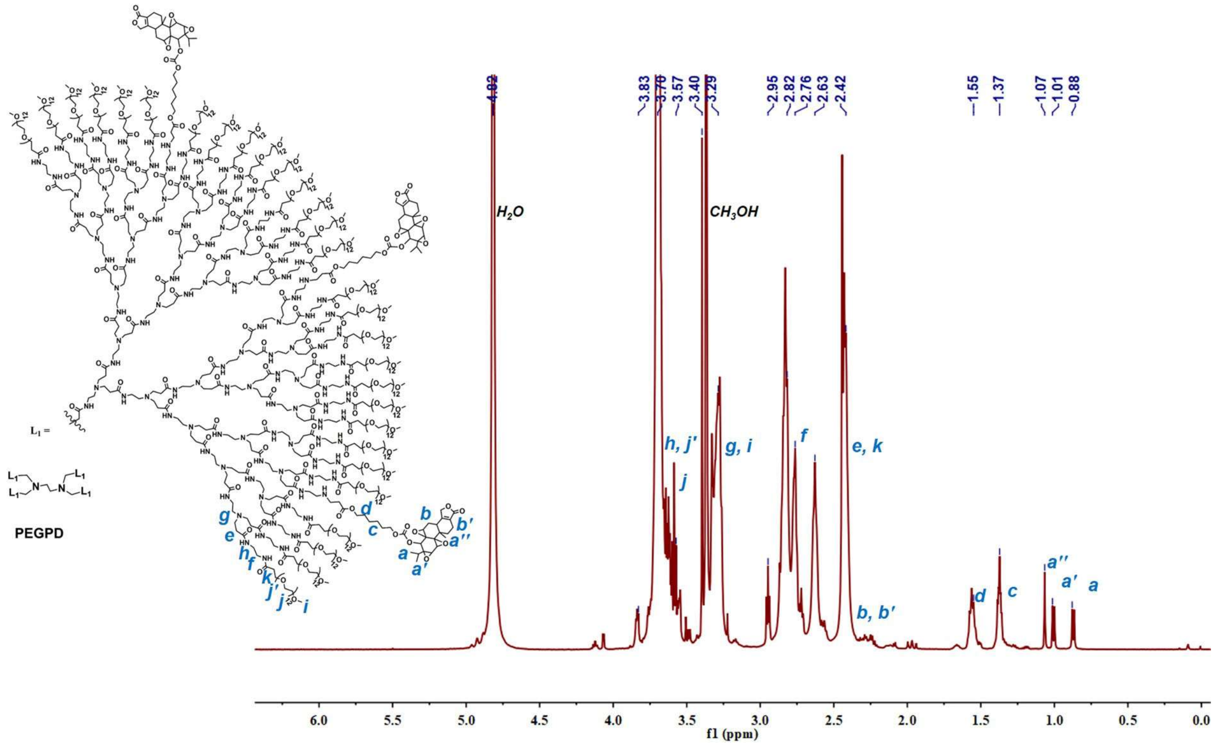

Figure S7. The ^{1}H NMR spectrum of PEGPD.

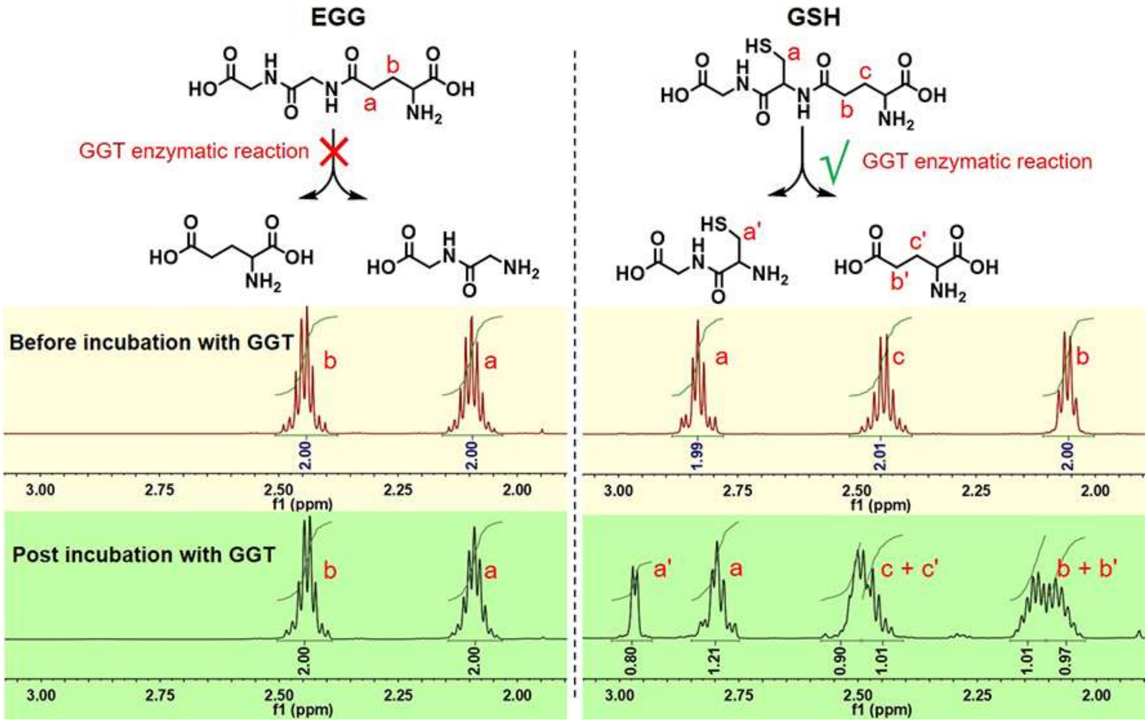

Figure S8. Structural changes of EGG and GSH on the ^{1}H NMR spectrum. EGG or GSH (10 mM) were incubated with GGT (10 U/mL) in PBS solution (pH = 7.4, prepared using D_2O) at 37°C for 4 h. The structural changes in EGG and GSH were detected using an Agilent DD2-600 MHz NMR spectrometer.

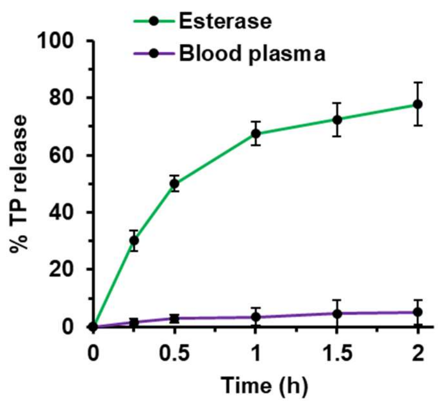

Figure S9. The percent of TP releasing from the GSHPD (equivalent to TP dose of 0.5 mg/mL) after incubation with rat blood plasma (total protein concentration about 60 mg/mL) and porcine liver esterase (200 U/mL, dissolved in the 100 mM, pH 7.4 Tris-HCl buffer) at different time points.

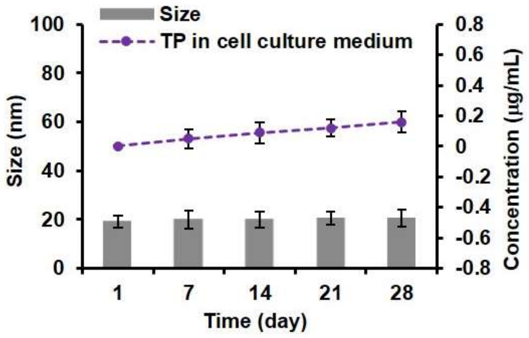

Figure S10. The stability assay of GSHPD in cell culture medium. The changes of particle size and TP content were respectively tested using DLS and HPLC after storage in different times of 1-day, 7-day,14-day, 21-day, 28-day at 25°C in the dark condition. The cell culture medium was comprised of DMEM medium containing 10% FBS, 100 U/mL penicillin, 100 μg/mL streptomycin, and added 5 mg/mL GSHPD (equivalent to the TP dose of 0.33 mg/mL).

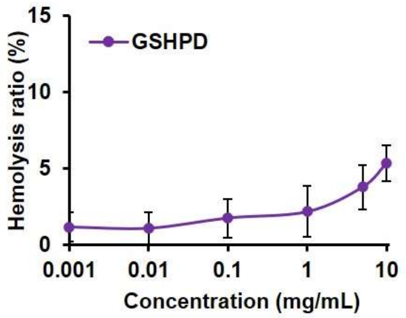

Figure S11. The hemolysis analysis of GSHPD in red blood cells. The erythrocytes were obtained from anticoagulated fresh rat blood. The erythrocytes were washed three times with

PBS solution (pH 7.4) via centrifugation at 2000 r/min for 5 min and then resuspended in PBS to obtain the erythrocyte stock solution with a concentration of 10^8 cells/mL. Subsequently, 0.25 mL of GSHPD suspensions with varying concentrations were mixed with 0.25 mL of erythrocyte stock solution in centrifuge tubes and incubated for 1 h in a 37°C shaker (60 r/min). The intact red blood cells were obtained from the pellet fraction after centrifuging at 2000 r/min for 5 min. The absorbance of the supernatant at 540 nm was measured to detect the released hemoglobin. PBS was added and served as negative control, and the mixture was treated with ultrasonication for fragmentation and served as positive control, respectively.

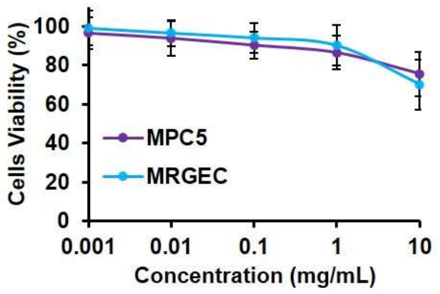

Figure S12. The toxicity assay of GSHPD in renal cells of MPC5 and MRGEC. The renal cells were treated with GSHPD in different concentration for 24 h. The cells viability was tested using the Cell Counting Kit-8 (CCK8, Beyotime Biotechnology, China).

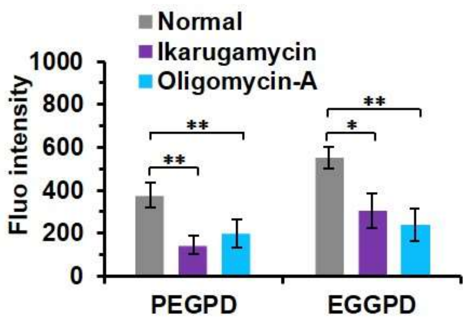

Figure S13. The inhibitory effects of clathrin inhibitor and ATP inhibitor on cellular uptake of EGGPD and PEGPD. The cell culture was respectively pretreated with clathrin inhibitor of Ikarugamycin (5 μg/mL) or ATP inhibitor of Oligomycin-A (1 μg/mL) for 2 h, and then replaced with fresh culture medium and added with conjugates for 1 h incubation. The MPC5

cells were collected, dispersed, suspended in 0.5 mL of PBS, and detected by flow cytometry. Comparison between the two indexed groups: $^{*}P < 0.05$, $^{**}P < 0.01$.

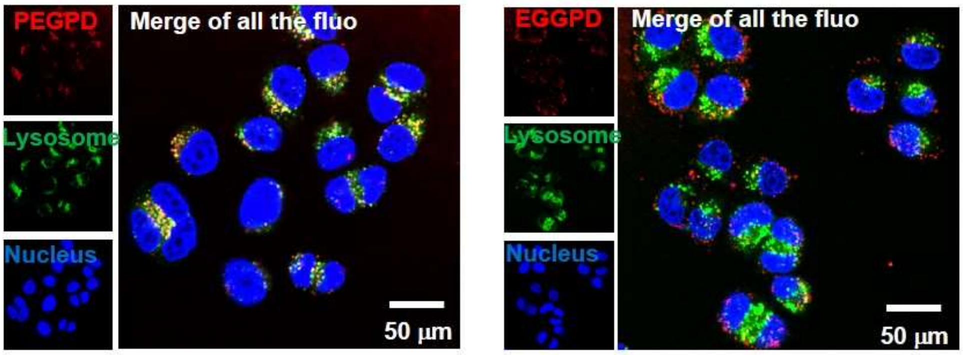

Figure S14. Subcellular distribution of PEGPD or EGGPD after 2 h incubation in MPC5 cells. The images were visualized using CLSM, as simultaneously stained nuclei (blue) using Hoechst 33342 and lysosomes (green) using LysoTracker Green DND-26 (Scale bar = 50 μm).

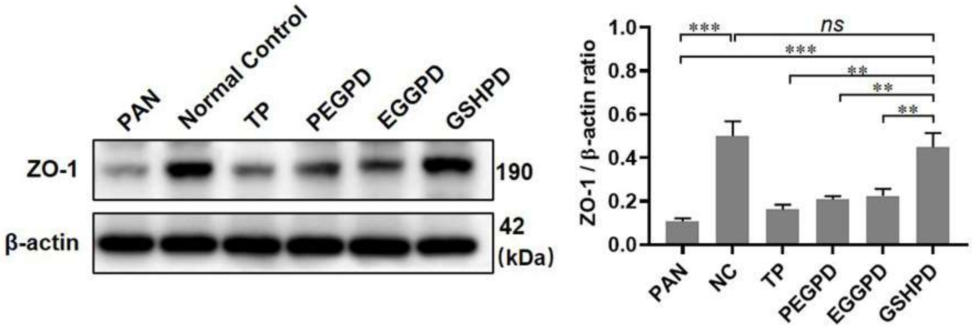

Figure S15. The effects of conjugates on ZO1 expression in podocyte injury model. The podocyte injury model was established using PAN (50 μg/mL)-treated MPC5 for 12 h at serum-starved culture condition. The cell culture was replaced with complete medium, and added with TP or conjugates (equivalent to TP dose: 2 μg/mL) for 24 h incubation. The cells were then collected, lysed, and quantitated for Western blotting assay using the anti-ZO1 tight junction protein antibody (#ab96587, Abcam) and the β-actin monoclonal antibody (#66009-1-Ig, Proteintech Group). Comparison between the two indexed groups: *ns* means no significance, $^{*}P < 0.05$, $^{**}P < 0.01$, $^{***}P < 0.001$.

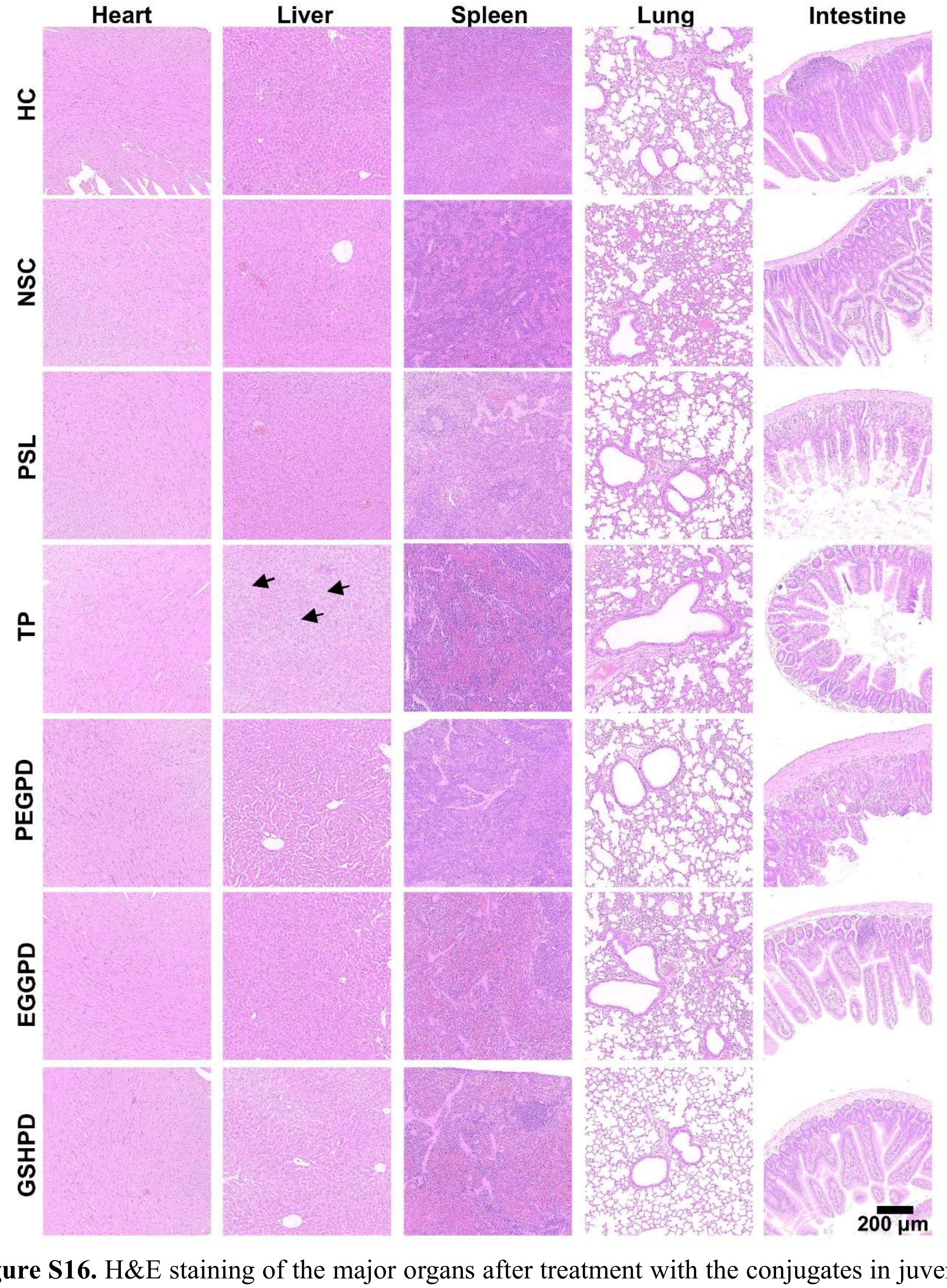

Figure S16. H&E staining of the major organs after treatment with the conjugates in juvenile rats with NS (Scale bar = 200 μm).

Table S1. The serum biochemistry analysis of juvenile rats with NS after treatments with the conjugates. The testing index of normal limits were referred to the SD rats (3~7 weeks of age) hematology in Charles River Laboratories of USA.

Groups/Testing index	ALT (IU/L)	AST (IU/L)
**HC**	33.6 ± 4.7	107.1 ± 9.2
**NSC**	38.3± 12.5	122.7 ± 11.0
**PSL**	42.1 ± 9.7	155.0 ± 18.4
**TP**	97.7 ± 16.2	242.4 ± 55.8
**PEGPD**	56.5 ± 12.0	188.2 ± 22.6
**EGGPD**	52.9 ± 14.4	157.2 ± 25.5
**GSHPD**	45.4 ± 10.1	134.0± 15.8
**Normal limits**	27~ 35	94 ~ 116
